# Inhibition of FGF2-Mediated Signaling in GIST—Promising Approach for Overcoming Resistance to Imatinib

**DOI:** 10.3390/cancers12061674

**Published:** 2020-06-24

**Authors:** Sergei Boichuk, Aigul Galembikova, Ekaterina Mikheeva, Firuza Bikinieva, Aida Aukhadieva, Pavel Dunaev, Dinar Khalikov, Semen Petrov, Refat Kurtasanov, Elena Valeeva, Igor Kireev, Vera Dugina, Anna Lushnikova, Maria Novikova, Pavel Kopnin

**Affiliations:** 1 Department of Pathology, Kazan State Medical University, 420012 Kazan, Russia; ailuk000@mail.ru (A.G.); miheeva.1973@bk.ru (E.M.); firuza1995@mail.ru (F.B.); arom1705@mail.ru (A.A.); dunaevpavel@mail.ru (P.D.); semyonp@mail.ru (S.P.); 2 Central Research Laboratory, Kazan State Medical University, 420012 Kazan, Russia; vevaleeva@ya.ru; 3 Tatarstan Cancer Center, 420029 Kazan, Russia; alik1x08@rambler.ru (D.K.); kurt-asan@mail.ru (R.K.); 4 Department of Electron Microscopy, A.N. Belozersky Institute of Physico-Chemical Biology, Lomonosov Moscow State University, 119992 Moscow, Russia; kireev@genebee.msu.ru; 5 V.I. Kulakov National Medical Research Center for Obstetrics, Gynecology, and Perinatology, 117997 Moscow, Russia; 6 Department of Mathematical Methods in Biology, A.N. Belozersky Institute of Physico-Chemical Biology, Lomonosov Moscow State University, 119992 Moscow, Russia; vdugina@iname.com; 7 Oncogenomics Laboratory, Carcinogenesis Institute, N.N. Blokhin National Medical Research Center of Oncology, 115478 Moscow, Russia; lan21@yandex.ru; 8 Cytogenetics Laboratory, Carcinogenesis Institute, N.N. Blokhin National Medical Research Center of Oncology, 115478 Moscow, Russia; mvnovikova94@mail.ru (M.N.); pbkopnin@mail.ru (P.K.)

**Keywords:** gastrointestinal stromal tumors (GISTs), imatinib (IM), sunitinib (SU), resistance, receptor tyrosine kinase (RTK), c-KIT and FGFR-signaling, FGF-2, autocrine pathway

## Abstract

Inhibition of KIT-signaling is a major molecular target for gastrointestinal stromal tumor (GIST) therapy, and imatinib mesylate (IM) is known as the most effective first-line treatment option for patients with advanced, unresectable, and/or metastatic GISTs. We show here for the first time that the inhibition of KIT-signaling in GISTs induces profound changes in the cellular secretome, leading to the release of multiple chemokines, including FGF-2. IM increased migration, invasion, and colony formation of IM-resistant GISTs in an FGF2-dependent manner, whereas the use of blocking anti-FGF2 antibodies or BGJ398, a selective FGFR inhibitor, abolished these effects, thus suggesting that the activation of FGF2-mediated signaling could serve as a compensatory mechanism of KIT-signaling inhibited in GISTs. Conversely, FGF-2 rescued the growth of IM-naive GISTs treated by IM and protected them from IM-induced apoptosis, consistent with the possible involvement of FGF-2 in tumor response to IM-based therapy. Indeed, increased FGF-2 levels in serum and tumor specimens were found in IM-treated mice bearing IM-resistant GIST xenografts, whereas BGJ398 used in combination with IM effectively inhibited their growth. Similarly, increased FGF-2 expression in tumor specimens from IM-treated patients revealed the activation of FGF2-signaling in GISTs in vivo. Collectively, the continuation of IM-based therapy for IM-resistant GISTs might facilitate disease progression by promoting the malignant behavior of tumors in an FGF2-dependent manner. This provides a rationale to evaluate the effectiveness of the inhibitors of FGF-signaling for IM-resistant GISTs.

## 1. Introduction

Gastrointestinal stromal tumors (GISTs) are the most common mesenchymal malignancies of the gastrointestinal tract arising from the specialized cells in the bowel wall, the interstitial cells of Cajal (ICCs), or their stem cell-like precursors. Since GISTs are frequently driven by the auto-activated, mutant KIT receptor tyrosine kinase gene or by the platelet-derived growth factor receptor alpha (PDGFR-α) [1,2,3], most of the tumors have a durable response to receptor tyrosine kinase inhibitor imatinib mesylate (Gleevec), which is currently considered as an effective drug for first-line therapy for GIST patients [4,5]. Unfortunately, despite the impressive response rates, more than 50% of patients with advanced and metastatic forms of the disease develop secondary resistance to imatinib (IM)-based therapy within 2 years after initiation of treatment [6,7]. To overcome secondary resistance to IM, more potent KIT inhibitors have been developed that are currently used as second- and third-line therapies for these patients. These include sunitinib malate (Sutent) [8] and regorafenib (Stivarga) [9], respectively. As KIT mutations are known as the most common mechanisms of secondary resistance to IM in GISTs, the alternative molecular mechanisms are also described and include hemi- or homozygous deletion of the wild-type KIT allele [10], overexpression of focal adhesion kinase (FAK) [11], amplification of the insulin-like growth factor receptor I (IGF-1R) [12], the BRAF V600E mutation (5% of GISTs) [13], and RTK “switch” (loss of c-KIT and gain of MET/AXL) [14]. We found recently that secondary resistance to IM in GISTs might also be due to the activation of the FGF-signaling pathway [15], whereas the inhibition of this pathway in IM-resistant GISTs effectively restores their sensitivity to IM both in vivo and in vitro [16]. This was in agreement with previous findings illustrating that IM-induced long-term KIT inhibition causes a feedback activation of FGFR-mediated signaling in GIST, thereby suggesting that FGFR inhibition may improve the long-term efficacy of IM in GIST patients [17]. Moreover, FGF-2 has been shown as a potent factor capable of desensitizing GIST to IM treatment, thereby providing insights into the protective role of FGF-signaling in IM-treated GISTs and illustrating the novel molecular mechanism of IM resistance for GIST patients lacking secondary resistance mutations in KIT [18]. Based on these findings, a phase Ib study was performed to evaluate the toxicity and dosing schedule of a combination of IM and BGJ398, a well-known selective FGFR-inhibitor. The stable disease for more than 32 weeks was observed in ~25% of heavily pretreated patients with IM-refractory GISTs (3/12). Unfortunately, toxicity was encountered with the combination therapy indicated above, and the trial was terminated before the dosing schedule of the combination therapy was determined [19].

We show here for the first time that IM induces profound changes in the cellular secretome of IM-resistant GISTs, leading to the release of multiple chemokines, including FGF-2, GRO, and MCP 1-3. Most importantly, IM promotes the malignant behavior of IM-resistant GISTs by stimulating their migration, invasion, and colony formation of tumor cells via the activation of the FGF-2/FGFR autocrine loop. IM-induced activation of FGF-signaling in IM-resistant GISTs was revealed in vivo by utilizing xenograft models and tumor specimens from GIST patients who have progressed after initiation of IM-based therapy. Altogether, this data suggests that the continuation of IM-based therapy for GIST patients with secondary resistance to IM might have a negative impact on disease progression due to the activation of FGF-signaling, thereby providing a rationale to evaluate the effectiveness of FGFR-inhibitors to improve the therapeutic strategies for IM-resistant GISTs.

## 2. Results

### 2.1. Inhibition of c-KIT Signaling Induces the Profound Changes in the Secretome of IM-Resistant GISTs

To assess the impact of the inhibition of KIT signaling on GIST secretomes, we initially utilized the IM-sensitive GIST T-1 cell line, which exhibits a heterozygous 57-base pair deletion (V570-Y578) in KIT exon 11 [20], and the IM-resistant GIST T-1R subline generated in our laboratory, as shown elsewhere [15]. The GIST 430 cell line exhibiting secondary resistance to IM due to a heterozygous secondary exon 13 missense mutation (V654A) was also utilized for the selected experiments [21].

**Figure 1:**
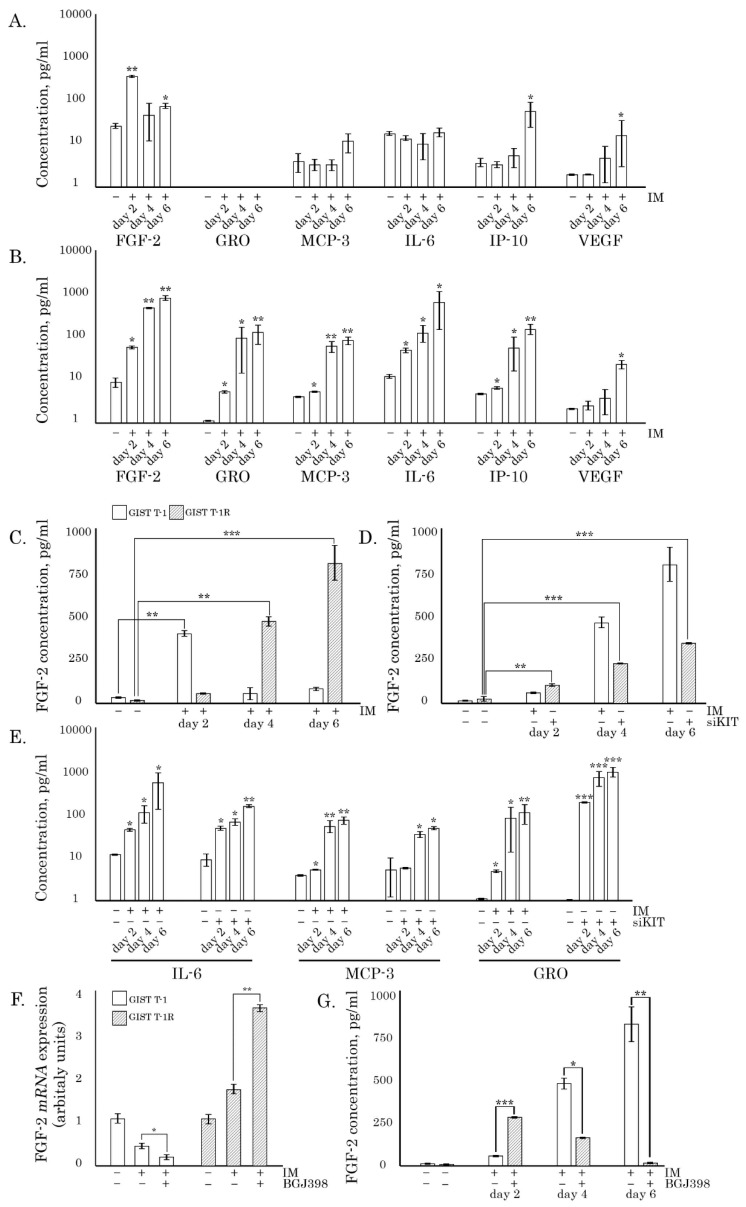
KIT inhibition induces the changes in gastrointestinal stromal tumor (GIST) secretome. (**A**) Median chemokine expression levels in the cell culture supernatants of imatinib mesylate (IM)-treated (1 µmol/L) GIST T-1 cells compared with non-treated controls. (**B**) Median chemokine expression levels in the cell culture supernatants of IM-resistant GIST T-1R cells treated with IM (1 µmol/L) compared with non-treated controls. (**C**) FGF-2 levels in supernatants of IM-naive vs. resistant GISTs treated with IM (1 µmol/L) for 2–6 days. (**D**) Comparative assessment of FGF-2 levels in cell culture supernatants of GIST T-1R cells treated with IM (1 µmol/L) or transfected with siRNA *KIT*. (**E**) Comparative assessment of IL-6, MCP-3, and GRO levels in cell culture supernatants of GIST T-1R cells treated with IM (1 µmol/L) or transfected with siRNA *KIT*. (**F**) Changes in the relative expression level of *FGF2* in GIST T-1 vs. T-1R cells treated with IM (1 µmol/L) alone or in the presence of BGJ398 (1 µmol/L), as determined by quantitative RT-PCR. For internal control, the amplification of glyceraldehyde-3-phosphate dehydrogenase (*GAPDH*) was used. (**G**) Comparative assessment of FGF-2 levels in cell culture supernatants of GIST T-1R cells treated with IM (1 µmol/L) alone or in the presence of BGJ398 (1 µmol/L). Data are presented as median ± SD. Significant differences with *p* < 0.05 (*), *p* < 0.01 (**), *p* < 0.001 (***) from n ≥ 3 using unpaired Student’s *t*-test.

The majority of cytokines/chemokines were undetectable in the supernatants of nontreated GISTs, whereas 6 cytokines (FGF-2, GRO, MCP-3, IL-6, IP-10, and VEGF) exhibited increased levels after IM treatment. The most significant changes were observed for FGF-2 and IP-10 in both IM-naive and -resistant GIST cells (Figure 1A,B, respectively), whereas the increase of IL-6 and GRO was specific for IM-resistant GISTs treated with IM. Of note, IM stimulated secretion of FGF-2 from IM-resistant (e.g., GIST T-1R) cells over the 6-day culture period in a time-dependent manner (Figure 1C), whereas in IM-naive GIST T-1 cells, the maximal FGF-2 levels were detected at day 2 of IM post-treatment and were further declined (Figure 1C), thereby reflecting IM-induced cell death of IM-naïve GIST T-1 cells. This was in consistency with the changes observed in the kinetics of growth (Figure S1A). In contrast to GIST T-1 cells, IM has no impact on the growth kinetics of both IM-resistant GIST cell lines utilized in the present study, GIST T-1R and 430 (Figure S1B,C, respectively). A similar FGF-2 pattern was observed for GIST T-1R cells transfected with siRNA *KIT*, thereby indicating the potential relationship between the inhibition of KIT-signaling and FGF-2 production (Figure 1D). Inhibition of KIT signaling in GIST T-1R cells was confirmed by Western blotting, which illustrated a substantial decrease of KIT phosphorylation in both IM- and siRNA *KIT*-treated GISTs (Figure S1D).

Similar to FGF-2, IL-6, MCP-3 and GRO were increased in supernatants of GIST T-1R treated with IM or siRNA *KIT*, thereby revealing the functional relationship between secretory phenotype and the inhibition of KIT-signaling (Figure 1E). This was in concordance with a real-time PCR data, illustrating an increase of mRNA *FGF-2* in IM-resistant GISTs after IM exposure, whereas IM treatment of IM-naive GIST T-1 cells substantially reduced mRNA *FGF-2* levels (Figure 1F). Interestingly, BGJ398, the FGFR kinase inhibitor, induced a significant (~6-fold) increase of FGF-2 levels in supernatants of IM-treated GIST-T1R cells on day 2 post-treatment (Figure 1G) and this fact correlated with an increase of mRNA *FGF-2* (Figure 1F), thereby indicating the possibility of the decreased consumption of FGF-2 produced by tumor cells after the inhibition of the FGFR-signaling pathway. As expected, in GIST T1-R cells treated with IM in the presence of BGJ398 for a longer period of time (for 4 and 6 days), the FGF-2 levels were significantly reduced due to the massive cell death in these experimental conditions (Figure 1G). Of note, IM-induced FGF-2 secretory pattern was also observed for GIST 430 cells exhibiting IM resistance due to the secondary *c-KIT* mutations (Figure S2A), thereby suggesting that IM-induced secretion of FGF-2 might be a common feature for IM-resistant GISTs.

Collectively, this data indicates that IM treatment of GISTs exhibiting signs of the activation of FGFR-signaling induces profound changes in GIST secretomes that might have a substantial impact on the motility, invasiveness and migration capacities of tumor cells.

### 2.2. Imatinib Stimulates Migration, Invasion, and Colony Formation of IM-Resistant GISTs in an FGF-2-Dependent Manner

**Figure 2:**
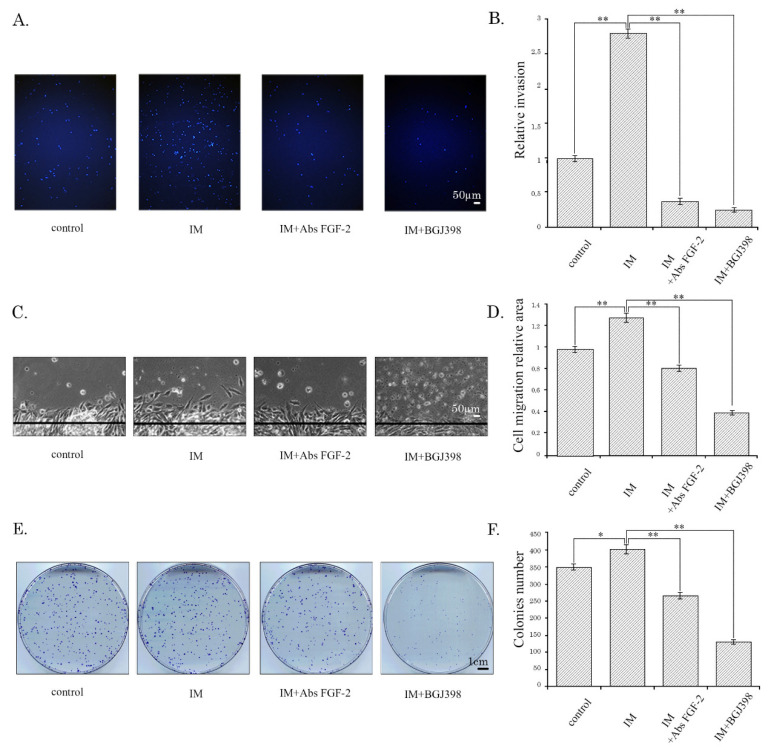
IM stimulates invasion, migration, and colony formation in GIST T-1R cells. (**A**) Matrigel transwell invasion assay representative images of GIST T-1R cells treated with vehicle, IM (1 µmol/L) alone or in the presence of BGJ398 (1 µmol/L), a selective FGFR inhibitor, or anti-FGF-2 neutralizing Abs (20 µg/mL). (**B**) Matrigel transwell invasion assay quantification as the number of invading GIST T-1R cells per microscopic field after treatment with vehicle, IM (1 µmol/L) alone or IM in the presence of BGJ398 (1 µmol/L) or anti-FGF-2 Abs (20 µg/mL). (**C**) Representative images of the wound healing assay of GIST T-1R cells upon IM treatment (1 µmol/L) for 48 h alone or in the presence of BGJ398, a selective FGFR-inhibitor (1 µmol/L) or anti-FGF2 neutralizing Abs (20 µg/mL). GIST cells treated with vehicle were used as control. (**D**) Quantitative analysis of wound area GIST T-1R cells treated with DMSO (control), IM alone or in presence of FGF-2 or BGJ398; (**E**) Representative images of the colony formation assay of GIST T-1R cells upon IM treatment (1 µmol/L) for 48 h alone or in the presence of BGJ398, a selective FGFR-inhibitor (1 µmol/L) or anti-FGF-2 neutralizing Abs (20 µg/mL). GIST cells treated with vehicle were used as a control. (**F**) Quantification of the colonies in GIST T-1R cells treated with DMSO (control), IM alone or in presence of FGF-2 or BGJ398. Data are presented as median ± SD. Significant differences with *p* < 0.05 (*), *p* < 0.01 (**) from *n* ≥ 3 using unpaired Student’s *t*-test.

To examine this possibility, the invasion and migration assays were performed on IM-resistant GIST cells pretreated with IM for 48 h. The scratch-wound healing assay was performed to examine tumor cell migration ability. The invasion of GIST cells was examined by the Transwell experiment, and the cells migrating through Matrigel-coated transwell chamber inserts were counted. FGF-2 was used as a positive control for this set of experiments and effectively stimulated the migration and invasion of IM-resistant GIST-T1R cells (Figure S2B–E, left panels). Strikingly, GIST T-1R cells treated by IM (1 μM), exhibited a substantial increase of invasion ability when compared to non-treated cells (*p* < 0.001; Figure 2A,B). To further delineate whether IM-induced activation of the FGF-2/FGFR autocrine loop is the mechanism through which IM regulates migration of GIST T-1R cells, we utilized the neutralizing anti-FGF2 Abs for IM-treated GIST cultures. Indeed, an increased invasion of IM-treated GIST T-1R cells was abolished by introducing the neutralizing anti-FGF2 Abs into the cell culture (Figure 2A,B). Similarly, when FGF-signaling was blocked by BGJ398, a selective FGFR inhibitor, the invasion ability of IM-treated GIST T-1R cells was substantially decreased (*p* < 0.001; Figure 2A,B).

We also observed that the migration of GIST cells at the edge of the scratch was enhanced following IM treatment when compared with the non-treated control. Again, the presence of anti-FGF-2 Abs or BGJ398 in cell culture remarkably decreased migration at the edge of the scratch in IM-treated GISTs. Similarly, BGJ398, a selective FGFR inhibitor, abolished this effect (Figure 2C,D).

The abilities of anti-FGF-2 Abs and BGJ398 to abolish the stimulatory effects of IM on IM-resistant GIST cells were also revealed by using a colony formation assay. As expected, the numbers of the colonies were modestly increased in IM-treated GIST T1-R cells when compared to non-treated cells and markedly reduced when GIST cells were treated with IM in the presence of BGJ398 or anti-FGF2 neutralizing Abs (Figure 2E,F).

Taken together, this data illustrates that besides IM being non-effective against IM-resistant GISTs exhibiting the activation of FGFR-signaling, this RTK inhibitor promoted migration, invasion, and proliferation of tumor cells. Important, these activities of IM could be interrupted by the inhibition of the autocrine FGF2/FGFR loop, thereby revealing that the IM-induced activation of the pathway indicated above might be a molecular mechanism underlying the aggressive phenotype of IM-resistant GISTs treated with IM and disease progression.

To delineate whether the increased migration and invasion is a common response of IM-resistant GISTs to IM treatment or if it is a specific feature of GIST T-1R subline, we assessed the effects of IM on the GIST 430 cell line exhibiting secondary resistance to IM due to a heterozygous secondary exon 13 missense mutation (V654A).

As expected, FGF-2 (a positive control) effectively stimulated invasion and migration of GIST 430 cells (Figure S2B–E, right panel). In contrast to GIST T-1R cells, IM has no significant impact on the invasion and migration capacities of GIST430 cells; however, FGFR inhibitor or anti-FGF-2 Abs significantly decreased both parameters indicated above in IM-treated GIST 430 cells (Figure S3A–D). Similar to GIST T-1R cells, we observed an increased number of colonies in IM-treated GIST 430 cells (Figure S3E,F). Again, the presence of anti-FGF-2 Abs or BGJ398, a selective FGFR1-4 inhibitor, abolished this effect of IM (Figure S3E,F). Strikingly, we also observed the large-size spherical colonies in IM-treated GIST 430 cells (Figure S3G,H), thereby suggesting that IM potentiates the growth of stem-like cell (SC) populations that form spherical clonal expanding colonies in this particular GIST cell line. More importantly, this effect of IM was abolished by BGJ398 or anti-FGF-2 (Figure S3G,H), thereby revealing IM-induced activation of the autocrine FGFR/FGF-2 loop in GIST 430 cells.

Altogether, this data illustrates that IM might promote the aggressive phenotype of GIST cells after they acquire secondary resistance to IM and thereby facilitate disease progression.

### 2.3. Interruption of Autocrine FGF-2/FGFR Loop Abrogates Activation of MAPK- but not AKT-Signaling Pathway in IM-Resistant GISTs

**Figure 3:**
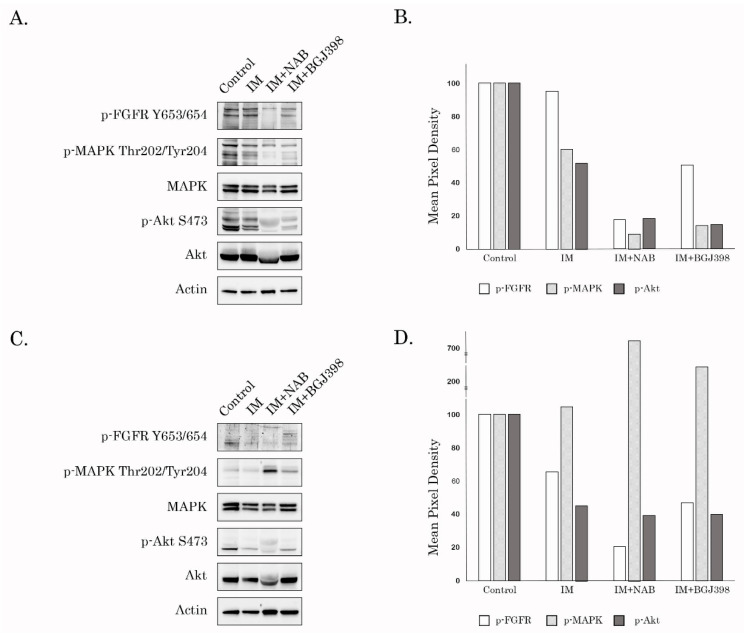
Inhibition of FGF-signaling deregulates AKT- and MAPK-phosphorylation in IM-resistant GISTs. (**A**) Anti-FGF2 neutralizing Abs (20 µg/mL) and BGJ398 (1 µmol/L) abrogate IM-induced activation of GIST T-1R cells. Cells were treated with IM (1 µmol/L) in the presence of anti-FGF-2 Abs for 72 h and subjected to the immunoblot analysis for total and phosphorylated forms of FGFR, MAPK, and AKT. Actin staining was used to show the comparable amounts of protein loaded into each sample. (**B**) Quantification by mean pixel density revealed that the phosphorylated proteins are dysregulated in GIST T-1R after the inhibition of FGFR signaling. (**C**) The impact of anti-FGF2 neutralizing Abs (20 µg/mL) and BGJ398 on IM-induced activation of GIST 430 cells. Cells were treated with IM (1 µmol/L) in the presence of anti-FGF-2 Abs for 72 h and subjected to immunoblot analysis for total and phosphorylated forms of FGFR, MAPK, and AKT. Actin staining was used to show the comparable amounts of protein loaded into each sample. (**D**) Quantification by mean pixel density revealed that the phosphorylated proteins are dysregulated in GIST 430 after the inhibition of FGFR signaling. The whole western blot of Figure 3A,C are in Figures S7 and S8.

To address the molecular mechanisms underlying the enhanced activities of IM-resistant GISTs after IM treatment, we assessed the activation of the downstream signaling cascades involved in KIT- and FGF-signaling pathways. We found that presence of anti-FGF-2 neutralizing Abs substantially decreased MAPK and AKT phosphorylation in IM-treated GIST T-1R cells (Figure 3A,B). Similarly, the expression of the phosphorylated form of FGFR1/2 was decreased when anti-FGF-2 Abs were introduced in IM-treated GIST culture. As expected, BGJ398, a selective FGFR inhibitor, provided similar effects on IM-treated GIST T-1R cells (Figure 3A,B). Similar to GIST T-1R, neutralizing anti-FGF-2 Abs decreased FGFR1/2 and AKT phosphorylation in GIST 430 cells (Figure 3C,D). However, IM-treated GIST430 treated with anti-FGF-2 Abs demonstrated elevated levels (up to ~6 fold) of activated MAPK, suggesting that GIST cells might compensate for AKT inhibition through increased MAPK signaling. Of note, a similar activation pattern was observed for MAPK/AKT in GIST430 cells treated with the combination of IM and BGJ398 (Figure 3C,D).

Thus, autocrine activation of FGF-signaling in IM-treated GISTs exhibiting secondary resistance to IM stimulates (e.g., GIST T-R) or maintains (e.g., GIST 430) their aggressive phenotype, resulting in the enhanced invasion and migration, and the activation of the downstream signaling pathways regulating cell proliferation and survival of tumor cells.

### 2.4. A Crosstalk between KIT and FGFR1/2 in IM-Resistant GISTs

Given that IM-induced inhibition of c-KIT-signaling activates the FGF2/FGFR autocrine loop in IM-resistant GIST cell lines, we sought to examine the possibility of whether KIT and FGFR1 and/or -2 interact directly or indirectly via common downstream signaling molecules. For this purpose, we performed immunofluorescence staining and co-immunoprecipitation (direct vs. reverse) between KIT and FGFR1/2 in IM-resistant GISTs.

**Figure 4:**
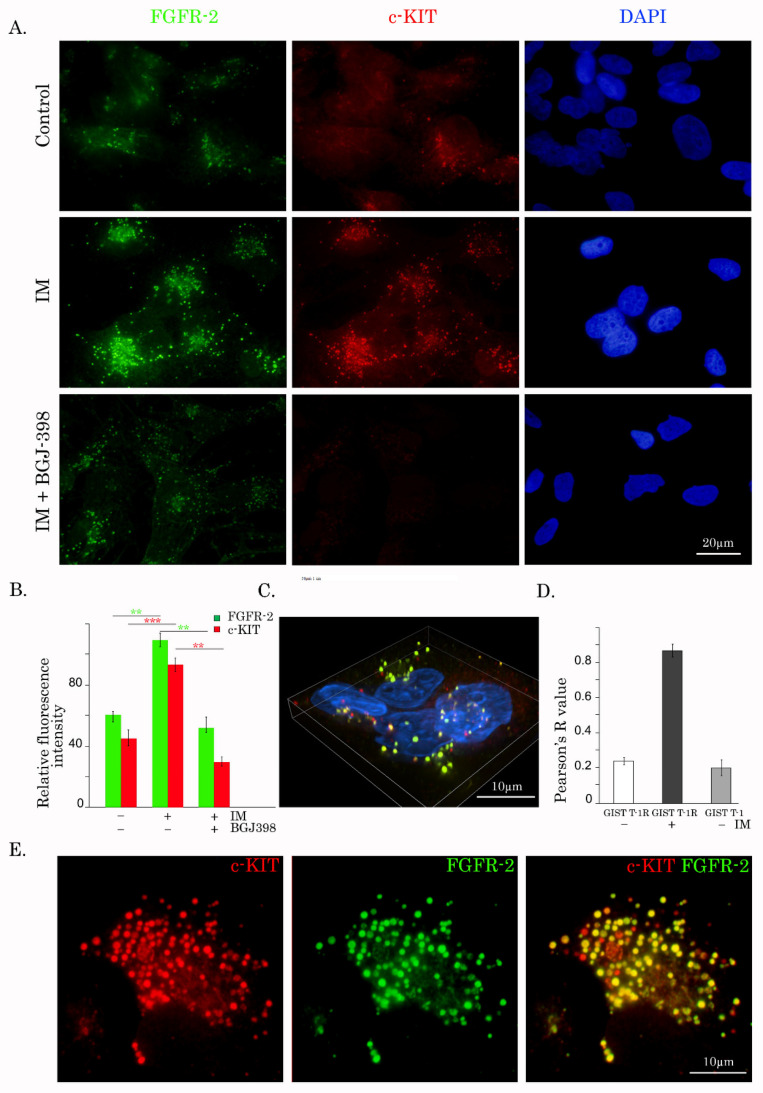
Crosstalk between FGFR2 and KIT in IM-resistant GISTs. (**A**) IM-resistant GISTs were treated with IM (1 µmol/L) for 48 h alone (middle panel) or in the presence of BGJ398 (1 µmol/L), a selective FGFR inhibitor (bottom panel), and subjected to the double immunofluorescence staining for FGFR2 and KIT. To outline the nucleus, the images were also merged with DAPI staining. (**B**) Quantitative analysis of relative fluorescence intensity of FGFR2 and KIT in GIST T-1R cells treated for 72 h with DMSO (control), IM (1 µmol/L) alone, or in the presence of BGJ398 (1 µmol/L). (**C**,**E**) Confocal images illustrating the co-localization pattern between FGFR2 (green) and KIT (red) in GIST T-1R cells. Merged image is shown in yellow in (**E**). Images were also merged with DAPI staining in (**C**) to outline the nucleus. (**D**) Pearson’s correlation analysis (Pearson’s R) for FGFR2 and c-KIT fluorescence pattern in IM-treated, control, and IM-naive cells is shown.

Although we failed to confirm the possible interactions between the endogenous KIT and FGFR1/2 in IM-resistant GISTs by co-immunoprecipitation experiments (Figure S4, the whole western blot of Figure S4 is in Figure S9), we observed a strong co-localization pattern between c-KIT and FGFR2 in GIST T1-R (Figure 4A–E), thereby suggesting the active crosstalk between these RTKs in IM-resistant GISTs.

Most importantly, the expression of FGFR2 was significantly increased in IM-treated GIST T-1R cells (Figure 4A—middle panel, B), revealing the activation of FGFR2-mediated signaling in KIT-inhibited GISTs. Of note, when tumor cells were treated with IM in the presence of BGJ398, expression of both types of RTK indicated above was decreased (Figure 4A—bottom panel,B). In addition to a quantitative difference between the mean values of staining for non-treated vs. IM-treated cells, FGFR2 and c-KIT dots were generally distributed equally in IM-treated cells. Moreover, IM induced co-localization between c-KIT and FGFR2, as shown by wide-field 3D-IF microscopy with deconvolution (Figure 4E). The fraction of c-KIT that co-localized with FGFR2 was measured via the Pearson’s overlap coefficient (also called Pearson’s R). The differences in co-localization between FGFR2 and c-KIT in IM-treated cells compared with untreated control cells and IM-naive cells are shown in Figure 4F. The R-value for IM-treated cells was above 0.70 (very strong positive relationship or co-localization), whereas the R-value for control and IM-naive cells had a weak positive relationship.

Similar to FGFR2, FGFR1 and KIT were co-localized in IM-treated GIST T-1R cells (Figure S5A–C). In addition, chemical inhibition of FGFR-signaling by BGJ398 in IM-treated GISTs reduced expression of RTKs measured by immunofluorescence staining (Figure S5A). As expected, we observed the decreased co-localization between FGFR1 and c-KIT after the combined treatment with IM and BGJ398 (Figure S5D).

### 2.5. FGF-2/FGFR Signaling Modulates GIST Responses to KIT Inhibition

**Figure 5:**
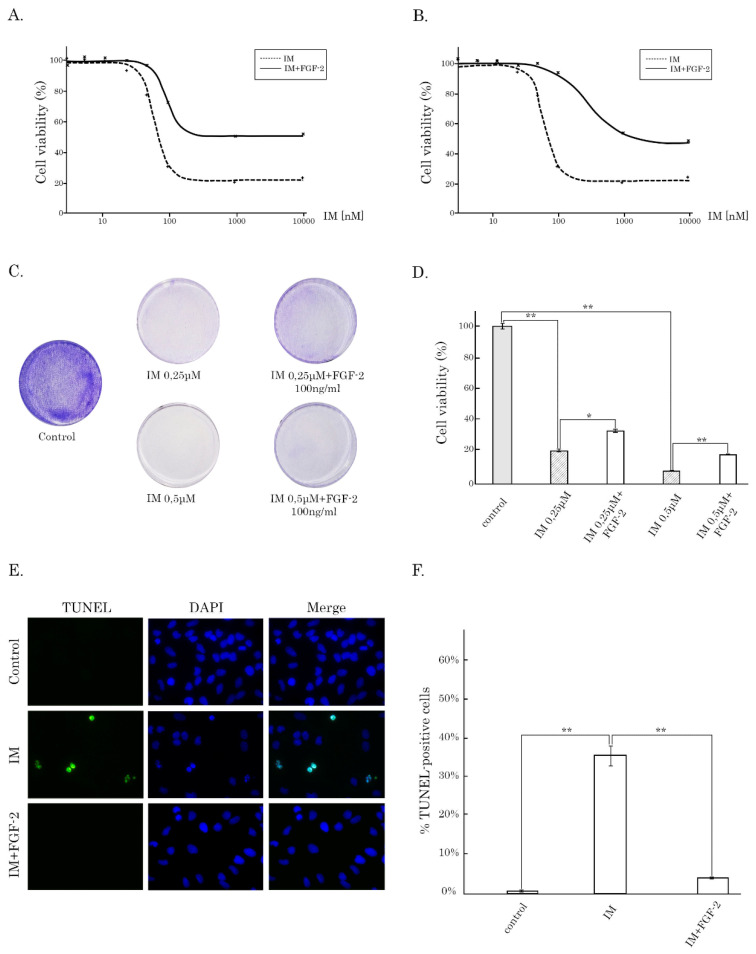
Activation of FGF-signaling rescues IM-naïve GISTs from KIT inhibition. (**A**) MTS-based cellular viability assay in GIST T-1 cells treated with IM alone for 48 h or in presence with FGF-2 (100 ng/mL). (**B**) MTS-based viability assay in GIST T-1 cells non-treated or pretreated with FGF-2 (100 ng/mL) for 14 days and further treated with IM for 48 h. (**C**) Crystal violet staining of GIST T-1 cells that were treated with IM alone or IM in the presence of FGF-2 for 72 h. The cells treated with DMSO were used as a control. The culture dishes were fixed with ice-cold 100% methanol, stained with crystal violet, and photographed. (**D**) Quantification of crystal violet staining of GIST T-1 cells, as shown in Figure 5C. (**E**) TUNEL-staining (40×) of GIST T-1 cells either mock-treated with DMSO or treated with IM (1 μmol/L) alone or in the presence of FGF-2 (100 ng/mL) for 72 h. DAPI was used to stain the nuclei. (**F**) Quantification of the percentage of TUNEL-positive GIST T-1 cells treated with DMSO (control), IM alone, or in the presence of FGF-2, as shown above. Columns, mean of at least 3 independent experiments with at least 100 cells calculated per experiment; bars, standard error (SD). Significant differences with *p* < 0.05 (*), *p* < 0.01 (**) from *n* ≥ 3 using unpaired Student’s *t*-test.

Given that IM-treated GIST T-1R cells actively produce FGF-2 (Figure 1C) and taking into account that the inhibition of FGFR-signaling restores their sensitivity to IM [15,16], we sought to examine whether the activation of FGFR-signaling might be a mechanism that rescues IM-naïve GIST T-1 cells from IM treatment. Indeed, we observed a ~1.5-fold difference in the IC50 values between IM-naïve GISTs treated with IM in the presence or absence of FGF-2 (93.5 ± 5.2 and 61.9 ± 2.7 nM, respectively; Figure 5A). Of note, pre-incubation of GISTs with exogenous FGF-2 for 2 weeks prior IM treatment substantially (e.g., 4.6-fold) enhanced GIST viability when compared to the experimental settings shown before—285.2 ± 46.3 and 61.9 ± 2.7 nM, respectively; Figure 5B). The crystal violet staining of GIST T-1 cultures revealed that FGF-2 effectively rescued the proliferation of tumor cells treated with IM (Figure 5C,D). This was also confirmed with a real-time cell proliferation assay using the iCELLigence system (Figure S6). As expected, IM (0.25 μM) has a potent anti-proliferative effect on GIST T-1 cells, whereas a substantial increase of proliferation was observed for the combination of IM and exogenous FGF-2 (100 ng/mL). Of note, exogenous FGF-2 used alone has a moderate proliferative effect, suggesting that survival and proliferation of KIT-mutant GIST cells are not initially dependent on FGF-signaling, and this pathway becomes important in GIST only after KIT inhibition. The last one is in close consistency with the data published by Li F. and coauthors, illustrating that BGJ398 had no effect on GIST cell growth as a single agent, but substantially potentiated the growth inhibitory effect of IM in both IM-naïve GIST T-1 and 882 cell lines (17). The ability of exogenous FGF-2 to protect IM-naive GIST T-1 cells from IM treatment was also revealed by a substantial decrease of apoptotic (i.e., TUNEL-positive) cells in GIST cultures treated with IM in the presence of FGF-2 when compared to GIST cells treated with IM alone (Figure 5E,F).

Collectively, our data illustrate that the activation of the FGF-2-based autocrine loop in GISTs is a potent mechanism modulating GIST responses to KIT inhibition and maintaining resistance to IM.

### 2.6. Increased Expression of FGF-2 in Tumor Tissues and Serum Post-IM Treatment

**Figure 6:**
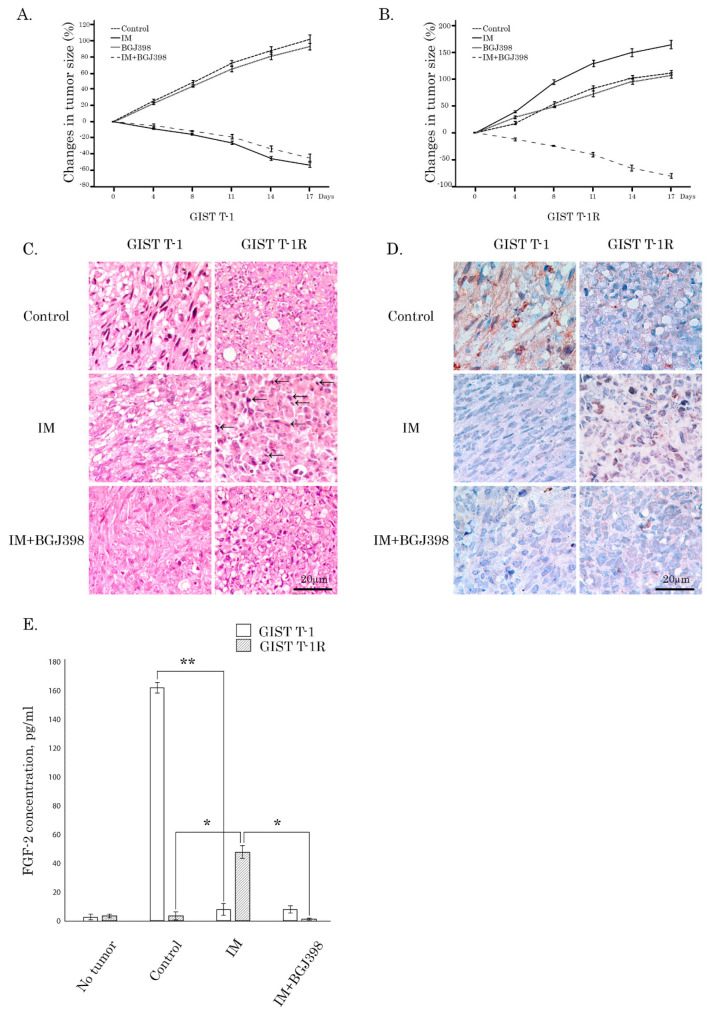
IM activates FGF-signaling in IM-resistant GIST xenografts. (**A**,**B**) Changes in the tumor volume of IM-naive (T-1) and resistant (T-1R) xenografts (A and B, respectively). Nude mice bearing GIST T-1R xenografts were orally administered with 50 μL of IM (50 mg/kg) or BGJ398 (60 mg/kg) alone or in combination. Mice treated with the same volume of the vehicle were used as a negative control. GIST T-1R xenografts were also treated with IM alone or in the presence of BGJ398. The changes in tumor size were calculated as a percentage of the baseline. GIST T-1R cells were maintained in culture in the presence of IM (1 μmol/L) for at least 1 month before injected into the flank areas of the nude mice. (**C**) Representative images of hematoxylin and eosin-stained IM-naïve (left) and -resistant (right) GIST xenografts treated for 7 days with IM alone (50 mg/kg) (middle panel) or in presence of BGJ398, a selective FGFR inhibitor (60 mg/kg) (bottom panel). Mitotic cells are depicted with arrows. (**D**) Representative images of FGF-2 immunohistochemical (IHC)-staining of IM-naive (left) and resistant (right) GIST xenografts non-treated (upper panel) and treated with IM alone (middle panel) or in the presence of BGJ398 (bottom panel). (**E**) FGF-2 serum levels in mice bearing IM-naive and -resistant GIST xenografts mock-treated and treated with IM (50 mg/kg) for 5 days. Columns, mean of at least 3 independent experiments; bars, SD. Significant differences with *p* < 0.05 (*), *p* < 0.01 (**) from *n* ≥ 3 using unpaired Student’s *t*-test.

To corroborate these findings, we examined the expression of FGF-2 in IM-naive vs. resistant GIST xenografts pre- and post-treatment of IM. For this purpose, GIST T-1 vs. T1-R cells were injected into the flank areas of female adult athymic nude mice, and the xenografts were allowed to grow for at least 14 days prior to IM treatment. As expected, IM substantially and time-dependently inhibited the growth of IM-naïve xenografts (Figure 6A), whereas most of the IM-resistant xenografts did not respond to this RTKi and exhibited a continuous increase in tumor size over the 2-week period of IM treatment when compared to the baseline (Figure 6B), thus revealing IM’s ability to promote the growth and invasiveness of IM-resistant GISTs. This was consistent with a substantial increase of mitotic cells observed after IM treatment of IM-resistant, but not IM-naïve GIST xenografts (Figure 6C, middle panel, as shown in arrows). When BGJ398 was used alone, no changes in tumor size were observed in GIST T-1R xenografts when compared to non-treated controls. Strikingly, when IM treatment of GIST T-1R xenografts was supplemented with BGJ398 treatment, a substantial decrease of tumor size was found (Figure 6B). Moreover, 8 of 10 GIST T-1R xenografts were completely resolved in this experimental group at the end-point of the combined treatment (IM plus BGJ398). Therefore, the inhibition of FGF-signaling was found to be extremely effective in our experimental xenograft model utilizing IM-resistant GIST T-1R cells lacking secondary KIT mutations.

IM-naive GIST xenografts exhibited a strong FGF-2 staining pattern, which was observed predominantly in the cytoplasm and decreased after IM treatment (Figure 6D, left panel). Conversely, FGF-2 expression in non-treated IM-resistant GISTs was relatively low and substantially increased on day 5 of post-IM treatment (Figure 6D, right panel). Moreover, this was associated with nuclear translocation of FGF-2, thereby suggesting the potential interaction of the ligand with its nuclear targets, promoting cancer cell proliferation, growth, and invasion. In concordance with this data, we found a significant increase of FGF-2 levels in the serum of IM-treated mice bearing IM-resistant GIST xenografts (Figure 6E), thereby revealing the activation of the autocrine FGF-2/FGFR loop in IM-resistant GISTs after initiation of IM-based therapy. Conversely, IM treatment of IM-naïve xenografts led to a substantial decrease of FGF-2 levels in serum (Figure 6E), which is consistent with the IHC data shown above. Incorporation of BGJ398 into an IM-based treatment schedule induced a substantial decrease of the numbers of mitotic and FGF-2-positive cells in GIST T-1R xenografts (Figure 6C,D, respectively), which correlated with the decrease of tumor size and volume in IM-resistant xenografts, thereby revealing that the activation of FGF-signaling is important for acquired resistance to IM in vivo. Of note, this was consistent with our previous data illustrating that this treatment schedule induced maximal histopathological scores, resulting in massive necrosis and myxoid degeneration [16]. Of note, BGJ398 used alone exhibited minor anti-tumor effects on IM-resistant xenografts (Figure 6B), thereby suggesting the activation of FGF-signaling as a compensatory mechanism of IM resistance in KIT-inhibited GISTs in vivo. Altogether, our data illustrate that interruption of FGF2-mediated signaling is highly effective against tumor cells exhibiting IM-induced activation of the autocrine FGF2/FGFR loop.

### 2.7. Increased FGF-2 Expression in GIST Specimens Post-IM Treatment

To corroborate the xenograft data, we examined FGF-2 expression in tumor specimens obtained from treatment-naive GIST patients (*n* = 20), and patients received IM-based therapy for at least 12–16 months (*n* = 10). The characteristics of GIST patients are presented in Table S1. Sixty-three percent of patients were female. The median patient age was 60 years. The most frequent location of GIST was the stomach (63%), followed by the small intestine (27%), rectum (3%), and unknown location/metastatic lesions (7%). 89% of tumors were positive for KIT IHC-staining. KIT/PDFRA mutational status was available for all GIST specimens analyzed in the present study: as expected, the most frequent were KIT mutations located in exon 11 (68%). According to the relapse risk criteria based on the AFIP classification, 41% of samples were considered as low risk and 59% as high risk. KIT/FGF-2 IHC-staining pattern in tumor specimens was analyzed and scaled according to the algorithm shown in Table S2. 

**Figure 7:**
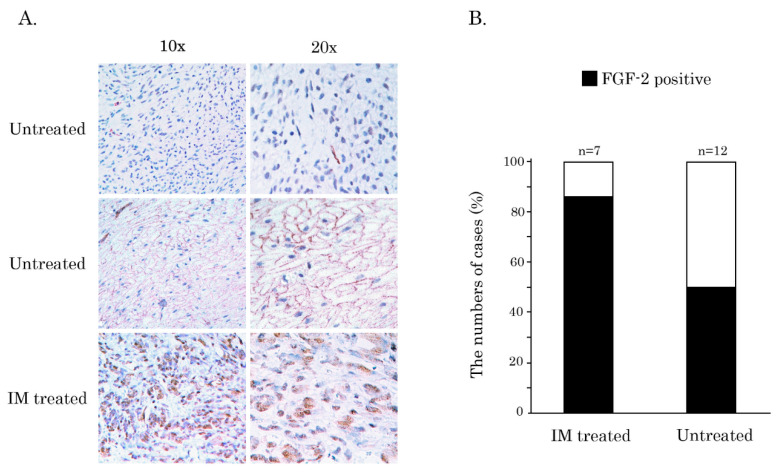
Overexpression of FGF-2 in GIST specimens after IM treatment. (**A**) Representative images of FGF-2 expression in primary non-treated vs. IM-treated GIST assessed by immunohistochemical (IHC)-staining of the tissue microarrays (TMA). Examples for low (upper panel), membranous (middle panel), and high nuclear (bottom panel) FGF-2 expression are shown in 3 GISTs at 10× and 20× magnification. (**B**) Graph depicting the numbers (in %) of FGF-2-positive GIST specimens (in the nucleus) in non-treated vs. IM-treated GIST patients. Only GIST samples exhibiting KIT/PDFRA mutations (as shown in Table S1) were included.

Although we failed to observe a correlation between FGF-2 expression and tumor location, KIT mutational status, gender and low/high-risk groups of treatment-naive GISTs (*p* > 0.05; Fisher’s exact probability test) or age (*p* > 0.05; ANOVA) and survival (*p* > 0.05; Kaplan–Meier’s test), we found the significant correlation between FGF-2 expression in GIST specimens and IM treatment (*p* = 0.01275; Fisher’s exact probability test). Indeed, 50% of non-treated GIST specimens stained for FGF-2 (*n* = 12) were negative for FGF-2 (Figure 7A—upper panel, B) or exhibited a weak cytoplasmic or membranous staining (Figure 7A, middle panel). Interestingly, the last one was specific for IM-naive GISTs exhibiting mutations in KIT exon 9. Strikingly, in the vast majority of IM-treated GIST specimens, we observed a strong nuclear staining pattern for FGF-2 (Figure 7A—bottom panel, B), illustrating the translocation of FGF-2 into the nucleus and the activation of FGF-signaling in GIST after IM treatment. This was in concordance with our xenograft data (Figure 6D, middle panel), thereby revealing the activation of autocrine FGF-2/FGFR-signaling and FGF-2 nuclear translocation in IM-treated GISTs in vivo. Given that nuclear localization of HMW FGF-2 triggers the activation of ERK1/2 (MAPK) [22] and regulates the activity of the genes that are maintaining the pro-survival cellular phenotype [23,24], this might be important for the survival of IM-treated GIST cells.

Taken together, our IHC data demonstrates a substantial increase of FGF-2 nuclear expression in GIST after the initiation of IM-based therapy (e.g., FGF-2 nuclear staining was observed in 6/12 cases (50%) of IM-naive GISTs and 6/7 cases (85.7%) of IM-treated GISTs (as shown in Figure 7B), thereby revealing the activation of FGF-signaling as a compensatory mechanism of IM resistance in KIT-inhibited GISTs in vivo. This is in close consistency with the previous findings illustrating that KIT inhibition in GISTs causes feedback activation of FGFR signaling in vitro [17,18].

Collectively, our data illustrate that IM induces the activation of FGF-signaling in GISTs in vitro and in vivo and this might have a significant impact on the malignant behavior of IM-resistant GISTs and disease progression. Further studies in larger cohorts of primary (i.e., treatment-naive) GISTs are needed to reveal the activation of FGF-signaling as a prognostic factor in primary GISTs.

## 3. Discussion

Fibroblast growth factors (FGFs) and their receptors (FGFR1–4) are known to be involved in the regulation of a wide spectrum of physiologic cellular processes, such as differentiation, proliferation, migration, survival, and angiogenesis. Meanwhile, aberrant FGFR-signaling is also well-documented for the broad spectrum of human malignancies and plays a regulatory role in tumor development and progression. Most importantly, the activation of FGF-signaling in tumors might mediate their sensitivity to the current therapeutic regimens, thereby affecting the effectiveness of anticancer therapies and disease prognosis.

In addition to well-documented FGFR mutations and/or *FGFR* amplifications leading to the activation of FGFR-signaling in multiple human malignancies, aberrant FGFR-signaling in cancer cells might be a result of the activation of autocrine/paracrine loops leading to increased secretion of FGFs by tumor or stromal cells and thereby promoting the survival and proliferation of cancer cells. The increased activities of autocrine and paracrine loops in human malignancies also play an important regulatory role in their pathogenesis due to the well-known abilities of FGFR ligands, especially FGF-2, to regulate angiogenesis and promote tumor growth, invasiveness, and disease progression. Moreover, in addition to the direct proangiogenic effects of FGFR-signaling, the activation of this pathway might have a strong impact on neoangiogenesis via the activation of VEGFR-signaling and thereby may synergize with VEGFR and platelet-derived growth factor receptor (PDGFR)-mediated pathways to promote tumor vascularization [25,26]. Activation of autocrine/paracrine FGF/FGFR-mediated loops was evidenced for multiple malignancies, including non–small cell lung carcinoma, hepatocellular carcinoma, and breast, prostate, and colorectal cancers [27,28,29]. The increased activity of FGF-2/FGFR autocrine/paracrine loops in malignancies is also considered as a potent mechanism of tumor resistance to conventional therapies, including targeted therapies [30,31].

However, to date, a little is known about the role of autocrine/paracrine FGF-2/FGFR-mediated signaling in GIST biology and their sensitivity to targeted-based therapy. For example, Li F. and coauthors demonstrated an increased expression of FGF-2 and FGFR-1 in the majority of primary GIST samples, thus suggesting the activation of FGF-2-mediated autocrine and/or paracrine loops [17]. Javidi-Sharifi N. and coauthors demonstrated that FGF-2 expression was increased in IM-resistant GIST cells, and KIT- and FGFR-3 inhibitors synergized to inhibit GISTs growth in vitro, thereby suggesting the crosstalk between KIT and FGFR3 in GISTs [18]. Consistent with these findings, our present data illustrates the activation of the FGF2/FGFR2 autocrine pathway in IM-resistant GISTs induced by c-KIT inhibition (by imatinib and siRNA, as well). In particular, we show here for the first time that KIT inhibition in GISTs induced the profound changes in GIST secretomes, reflecting a massive secretion of the multiple chemokines (e.g., MCP-3, GRO, IP-10, FGF-2, and VEGF) known to be elevated in many types of human cancers, and promoting cancer cell invasion and migration. In particular, we observed that FGF-2, the FGFR1-4 ligand, was actively produced by IM- or *siKIT*-treated GISTs (both of IM-naive and -resistant) in a time-dependent manner, thus suggesting the activation of the autocrine FGF-2/FGFR loop could serve as a compensatory mechanism of the c-KIT-signaling pathway inhibited in GISTs (Figure 1D). Activation of the FGF2/FGFR autocrine loop in IM-treated GISTs was also revealed by in vivo studies illustrating nuclear localization of FGF-2 in GIST xenografts after IM treatment (Figure 6D) and tumor specimens of GIST patients who received IM-based therapy (Figure 7A). The molecular mechanisms involved in FGF-2 trafficking into the nucleus in IM-treated GISTs remain to be further elucidated and might include its association with the cytoplasmic microtubule-associated protein translokin, FGF-2-interacting factor (FIF). The first one is known to be important for low molecular weight (18-kDa) FGF-2, harboring a C-terminal nuclear localization sequence (NLS) [32], whereas the second one regulates the nuclear trafficking of all the high molecular weight isoforms (22-, 22.5-, 24-, and 34-kDa) of FGF-2 containing N-terminal NLS [33].

Besides active angiogenesis and increased growth kinetics in tumors exhibiting the activation of FGF-2/FGFR autocrine and/or paracrine loops, activated FGF-signaling was shown as a potent regulator of migration and invasion of cancer cells via diverse molecular mechanisms [34,35,36,37,38]. Most of them reflect the paracrine loop-mediated mechanisms. For example, fibroblast cell surface-associated fibroblast growth factor (FGF)-2 was shown to induce the cell-contact-dependent migration and invasion of colorectal cancer cells via the activation of the FGF-2-FGFRs-SRC-αvβ5 integrin axis [37], whereas the activation of FGF-2/FGFR-1 paracrine signaling in breast cancer cells induces the expression of the connective tissue growth factor (CTGF), thereby promoting the migration and invasion of tumor cells [38].

Consistent with these findings, we showed here for the first time that increased production of FGF-2 by IM-treated GIST cells reflecting the activation of the FGF-2/FGFR autocrine loop can serve as a mechanism maintaining the aggressive behavior of IM-resistant tumor cells through the facilitation of their migration and invasion. Interruption of the FGF-2/FGFR signaling via neutralizing anti-FGFR-2 Abs abolished this effect, thereby revealing that IM-induced activation of the FGF-2/FGFR autocrine loop is responsible for increased migration and invasion of IM-resistant GIST T-1R cells. Similarly, BGJ398, a selective FGFR inhibitor, abolished IM-induced increase of migration and invasion of IM-resistant GISTs (Figure 2 and Figure S3). Of note, we also observed the large-size spherical colonies in IM-treated GIST 430 cells (Figure S3E,G), thereby suggesting that IM might potentiate the growth of the cells with stem-like cell (SC) properties. Again, this effect of IM was abolished by BGJ398 or anti-FGF-2 Abs (Figure S3E–H), thereby illustrating the IM-induced activation of the FGF2/FGFR autocrine pathway is not restricted to one particular GIST cell line. To validate the SC-like phenotype in GIST 430 after IM treatment, further experiments are needed, including the cell culture in specific (e.g., non-adherent) culture conditions, by utilizing ultra-low attachment surfaces and examination of markers specific for the CSC/progenitor population, such as aldehyde dehydrogenase (*ALDH1A1*) mRNA expression and ALDH activity and expression, as was shown before [39].

Thus, IM activates the FGF-2/FGFR autocrine loop in various IM-resistant GIST cell lines and this mechanism might have a negative impact on the disease progression due to the activation of tumor cell migration, invasion, and enrichment of tumors with stem-like cell (SC) properties.

Of note, IM-induced activation of the FGF-2/FGFR loop in GIST might be a factor mediating tumor resistance to IM. Indeed, an exogenous FGF-2 rescued IM-naive GIST cells from IM-induced apoptotic cell death (Figure 5E,F) and significantly (~4-fold) increased IC50 values of IM in IM-naïve GIST cells (Figure 5B). The last one is consistent with previous findings illustrating a protective role of FGF-2 in IM-naive GIST cell lines (GIST T-1 and 882) treated with the low doses of IM [18]. The anti-apoptotic activity of FGF-2 in GISTs might be due to the different molecular mechanisms. It was recently shown that the anti-apoptotic activity of FGF-1 and -2 is not solely due to the activation of FGFR-signaling (e.g., AKT- and MAPK-mediated pathways) and might be a result from the intracellular translocation of the ligands into the nucleus [40]. Indeed, besides the activation of the tyrosine kinase receptors, secreted FGF-1 and FGF-2 can also translocate across the cellular membranes into the cytosol upon internalization via FGFR-dependent endocytosis, and this process is tightly regulated by several cytosolic proteins, including heat shock protein 90 (HSP90), PI3K- and p38 kinase [36,37,38]. Nuclear import of FGFR ligands is more specific and depends on the interactions between FGF-1 and -2 with LRRC59 protein and translokin, respectively [32,41]. Despite the complexity of the mechanisms involved in FGF-1 and -2 internalization and intracellular trafficking, it is known that nuclear transportation of these ligands is frequently observed during various types of stress conditions, such as exposure to the toxic agents, oxidative stress, hyperosmolarity, and serum deprivation, thereby highlighting their protective role in the nucleus during the unfavorable conditions indicated above. Indeed, the nuclear translocation of FGF-1 was shown to promote cell survival via the downregulation of p53-dependent apoptosis [40,41,42]. Similarly, the antiapoptotic activity of intracellular FGF-2 was shown to be mediated via its binding to apoptosis inhibitor 5 (API5) [33]. Our data illustrates that FGF-2 is actively secreted by IM-treated and KIT-inhibited GISTs (Figure 1D), activates FGF-signaling via phosphorylation of FGFR1 and -2 (Figure 3) and translocates into the nucleus (Figure 6D and Figure 7A), thereby illustrating the variety of FGF-dependent mechanisms of GIST resistance to IM-induced apoptosis.

Taken together, IM-induced activation of FGF-signaling pathway in GISTs is in agreement with a paradigm indicating that FGFRs and their ligand, FGF-2, could work as a surrogate signaling pathway under the inhibition of RTK-signaling and mediate tumor resistance to RTK inhibitors (RTKi) and chemotherapeutic agents as well. For example, FGF-2-FGFR-1 activation through an autocrine loop was found as a mechanism of acquired resistance in non-small cell lung cancer (NSLC) to EGFR-tyrosine kinase inhibitor gefitinib [30,31]. Moreover, FGF-2 leaking out from gefitinib-treated NSLC cells was proposed as a survival factor for tumor cells that remained alive after the first exposure of EGFR-TKIs and further maintained their resistance to EGFR-TKIs [42,43]. FGF2-mediated tumor resistance to a broad spectrum of chemotherapeutic drugs with diverse structures and mechanisms of action is also well-documented. For example, Song S. et al. demonstrated that increased concentrations of bFGF in the conditioned medium of solid and metastatic tumors might mediate their resistance to certain chemotherapeutic agents—paclitaxel, doxorubicin, and 5-fluorouracil—whereas the inhibition of bFGF by monoclonal antibody abolished this effect [44]. Similarly, the elevated intracellular level of bFGF in CLL was associated with their resistance to chemotherapy and correlated with an advanced stage of the disease [45].

Most importantly, an increased FGF-2 level was found in plasma samples of patients affected by diverse malignancies, such as leukemia and lung and breast cancers, especially when metastases are present and therefore suggested increased FGF-2 levels in serum as a complement predictor of poor prognosis. For example, high serum levels of bFGF were found to be associated with poor outcomes in small cell lung cancer [46] and non-Hodgkin’s lymphoma [47]. In addition, high levels of FGF-2 in plasma were associated with large tumor size in head and neck cancers [48] and with tumor growth kinetics in advanced colorectal cancer [49]. Increased basic-FGF plasma concentrations are also correlated with high tumor grade and stage, metastatic spreading, and poor prognosis in kidney cancer patients, thereby illustrating that fibroblast growth factor receptor (FGFR) pathway is an attractive therapeutic target for patients with renal cell carcinoma [50].

Consistent with this data, we observed the elevated FGF-2 levels in serum of IM-resistant GIST xenografts treated with IM when compared to non-treated mice bearing IM-resistant GISTs (Figure 6E). Of note, FGF-2 levels in serum correlated with the xenograft volume and size, thereby suggesting a significant impact of autocrine FGF-2-mediated signaling for GIST behavior during IM treatment. Moreover, increased FGF-2 expression was also observed in IM-treated GIST xenografts, when compared to controls (Figure 6D, middle panel). IHC-staining also revealed a substantial increase of FGF-2 expression in GIST human specimens after IM-based therapy when compared to non-treated patients with primary GISTs (Figure 7A,B). Most importantly, this was also associated with the increased numbers of mitotic cells in GISTs after IM treatment (Figure 6C, middle panel), thereby revealing IM’s ability to promote aggressive phenotypes in GISTs during long-term therapy.

Overall, our present data illustrate that continuation of IM-based therapy might have a negative impact on GISTs’ behavior after the tumors acquire resistance to IM. This is due to IM-induced activation of the autocrine FGF-2/FGFR loop, thereby facilitating disease progression via stimulation of GIST proliferation, migration, and invasion. This, in turn, provides a rationale to further evaluate the effectiveness of FGFR-inhibitors to improve the therapeutic strategies for GIST patients with an acquired (i.e., secondary) resistance to IM.

## 4. Materials and Methods

### 4.1. Chemical Compounds

Imatinib mesylate (IM) and BGJ398 were purchased from SelleckChem (Houston, TX, USA).

### 4.2. Antibodies

Primary antibodies used for immunoblotting and immunofluorescence were as follows: phospho-MAPK (Erk1/2) Thr202/Tyr204 (#4370S), MAPK (Erk1/2) (#4696S), phospho-AKT S473 (#4060P), AKT (#4691p), phospho-FGFR Y653/654 (#3476S), FGFR1 (#9740S), FGFR2 (#23328S) phospho-KIT Y719 (#3391S) (Cell Signaling, Danvers, MA, USA), c-KIT (#A4502, Dako, Carpinteria, CA, USA), beta-actin (A00730-200, GenScript, Piscataway, NJ, USA). HRP-conjugated secondary antibodies used for Western blotting were from Santa Cruz (Santa Cruz Biotechnology, Santa Cruz, CA, USA). Primary antibodies used for IHC-staining were as follows: c-KIT (#14-1172-82, Thermo Fisher Scientific, Waltham, MA, USA), DOG-1 (#244R, Cell Marque, Rocklin, CA, USA), a cleaved form of caspase-3 (#9662S, Cell Signaling, Danvers, MA, USA) and FGF-2 (sc-365106, Santa Cruz Biotechnology, Santa Cruz, CA, USA). Neutralizing antibody against bFGF (Anti-FGF2/basic FGF #05-117) and human recombinant FGF-2/basic (FGF2 #01-106) were obtained from Merck KGaA (Darmstadt, Germany).

### 4.3. Cell Lines and Culture Conditions

The IM-sensitive GIST T-1 cell line was established from human metastatic pleural tumors arising from stomach GIST and exhibited a heterozygous 57-base pair deletion (V570-Y578) in *KIT* exon 11 [20]. The IM-resistant GIST T-1R subline was generated in our laboratory, as shown elsewhere [15]. IM-resistant GIST 430 cell line was established from human GIST that acquired clinical resistance to IM-based therapy. This GIST cell line exhibited a heterozygous primary *KIT* exon 11 deletion (V560_L576del) and a secondary *KIT* exon 13 point mutation (V654A) [21]. GIST cell lines indicated above were cultured in a humidified atmosphere of 5% CO_2_ at 37 °C (LamSystems, Russia), as previously described [16].

### 4.4. TUNEL Assay

To evaluate apoptotic cell death in GIST cells, the terminal deoxynucleotidyl transferase nick end labeling (TUNEL) assay was performed by using a commercially available in situ apoptosis detection kit (DeadEnd™ Fluorometric TUNEL System, Madison, WI, USA). TUNEL-positive cells were counted by using a ×40 objective on an Olympus BX63 fluorescence microscope.

### 4.5. Cellular Survival MTS-Based Assay

IM-naïve and -resistant GIST cell lines indicated above were plated on 96-well flat-bottomed plates (Corning Inc., Corning, NY, USA) and allowed to attach and grow for at least 24 h before treatment with RTK inhibitors (e.g., IM and BGJ398) that were introduced into the cell culture for 48–72 h with indicated concentrations alone or in combination with each other. For specific experiments, cells were treated with RTKis in the presence or absence of FGF-2. To calculate the IC50 values of the RTKi, MTS reagent (Promega, Madison, WI, USA) was introduced into the cell culture for at least 1 h to assess the live cell numbers. The cellular viability was assayed at 492 nm on a MultiScan FC plate reader (Thermo Fisher Scientific, Waltham, MA, USA). Resulting IC_50_ values were defined as the compound concentration required to inhibit cellular growth by 50%. This data was normalized to the DMSO-treated (i.e., control) cells.

### 4.6. Real-Time Monitoring of Cell Proliferation

The growth curves of GIST T1 cells cultured in the presence of IM, FGF2 alone, or in combination were analyzed by using the iCELLigence system (ACEA Biosciences, San Diego, CA, USA). For this purpose, tumor cells were plated on the electronic microtiter plates (E-Plate; Roche Diagnostics, GmbH, Mannheim, Germany) for at least 24 h and further treated with IM (0.25 µM), FGF-2 (100 ng/mL) alone or in combination for 72 h. The cells treated with solvent (DMSO) were used as a negative control. The cell index (CI) was measured every 30 min until the end time-point of the experiment (72 h). Normalized cell index (NCI) values were also analyzed by using RTCA software (Roche Diagnostics, GmbH, Mannheim, Germany).

### 4.7. Western Blotting and Coimmunoprecipitation (Co-IP)

To examine the protein expression in GIST cells, whole-cell lysates (WCL) were prepared by scraping the cells growing as monolayer into RIPA buffer (25 mM Tris-HCl pH 7.6, 150 mM NaCl, 5 mM EDTA, 1% NP-40, 1% sodium deoxycholate, 0.1% SDS), supplemented with the cocktail of protease and phosphatase inhibitors. The cellular lysates were further incubated for 1 h at 4 °C and clarified by centrifugation for 30 min at 13,000 rpm at 4 °C. The protein concentrations in WCL were calculated by the Bradford assay. The protein samples (30 μg) were loaded on the 4–12% Bis-Tris or 3–8% Tris-acetate NuPAGE gels (Invitrogen, Carlsbad, CA, USA), transferred to a nitrocellulose membrane (Bio-Rad, Hercules, CA, USA), probed with a specific antibody, and visualized by enhanced chemiluminescence (Western Lightning Plus-ECL reagent, Perkin Elmer, Waltham, MA, USA). Densitometric analysis of Western blotting images was performed by using NIH ImageJ software (Bethesda, MD, USA).

For Co-IP, GISTs were lysed by TEB buffer (50 mM Tris-HCl pH 7.5, 150 mM NaCl, 1% NP-40, 10% glycerol) and supplemented with the cocktail of protease and phosphatase inhibitors. The lysates were clarified by centrifugation, as shown above, and cultured with the specific Abs overnight at 4 °C in a rotating device. The next day, the samples were treated with protein A/G Sepharose beads (Santa Cruz Biotechnology, Santa Cruz, CA, USA) for 1 h (rotating device at 4 °C). Finally, the beads were washed with TEB buffer and the 1X samples were subjected for Western blotting, as indicated above.

### 4.8. Immunofluorescence Staining

Cells were seeded on glass coverslips pre-coated with poly-L-lysine (Sigma-Aldrich, St. Louis, MO, USA) and allowed to attach for 48 h before treatment. For immunofluorescence staining, the cells were washed with PBS, fixed in 2% paraformaldehyde for 10 min at room temperature, washed with PBS again, and additionally fixed with ice-cold methanol for 10 min. Methanol was removed with PBS washing (5 times for 5 min). Alternatively, washed cells were fixed in 4% paraformaldehyde in PBS for 30 min at 4 °C and permeabilized with 0.5% Triton X-100 for 5 min. The cells were blocked for 30 min by using 10% normal goat serum and further incubated with primary antibodies for overnight at 4 °C. The next day the cells were washed with PBS, incubated with Alexa Fluor 488 or TexRed-conjugated secondary antibodies (Invitrogen, Carlsbad, CA, USA) for 30 min at room temperature in the dark. Finally, a DAPI stain (Sigma-Aldrich, St. Louis, MO, USA) was used for 1 min to outline the nuclei; the coverslips were washed with PBS twice and mounted on glass slides. The cells were visualized on an Olympus BX63 fluorescence microscope. Images were captured using a Spot advanced imaging system or a Nikon N-SIM confocal system.

### 4.9. D-IF Microscopy (Wide-Field with Deconvolution)

Glass coverslips with cells after IF staining were mounted on PVA (polyvinyl alcohol, Sigma-Aldrich, St. Louis, MO, USA). Samples were examined using N-SIM microscopy (Nikon) with an EMCCD camera (iXon 897, AndorT). Then, 488 and 561 nm laser excitation and 100x/1.49 NA oil immersion objectives were used. Exposure conditions were adjusted to get a typical yield of about 5000 max counts (16-bit raw image) for minimal bleaching. Images have been captured as serial optical sections of the same cell in *z*-axis with 0.12 μm z-steps. For serial images in wide-field mode, the AutoQuant blind deconvolution algorithm was used. Image analysis, visualization, archiving, and volume views were performed using NIS-Elements 4.2 microscope imaging software (Nikon).

### 4.10. Colocalization Analysis

Co-localization of FGFR2 and c-KIT was performed on thresholded super-resolution images to exclude false-positives of the background generated by SIM reconstruction algorithms. Co-localization was analyzed by the Coloc 2 plugin included in the FIJI package, run on ROIs containing prominent features. Costes’s algorithm was applied for automatic threshold detection [51], and Pearson coefficients were calculated for the above-threshold pixels.

Co-localization analysis for FGFR1 and c-KIT was performed in FIJI Software (Laboratory for Optical and Computational Instrumentation, University of Wisconsin, Madison, WI, USA). The Parallel Spectral Deconvolution plugin was applied to restore the contrast of the images, and the degree of co-localization between FGFR1 and c-KIT was measured by using the Coloc2 plugin. Spearman’s rank correlation values of at least 40 cells per each experimental condition were calculated, and their means were compared by using the analysis of variance (ANOVA) with subsequent pairwise comparisons (*t*-test with Bonferroni adjustment) in R software (R Foundation for Statistical Computing, Vienna, Austria; URL https://www.R-project.org/).

### 4.11. Wound-Healing Assay (Scratch Assay)

Cells were pre-cultured with DMSO (control), and IM (1 µM) alone or in the presence of anti-FGF-2 mAbs (5 mg/mL) or BGJ398 (1 µM) for 72 h and further seeded in 6-well plates and the tip of a 200 μL micropipette was used to make a scratch on a confluent monolayer of cells to create a wound. The detached cells were rinsed with PBS and then DMEM-Hi medium was added. The wound area was photographed by an AxioVert 200 microscope (Carl Zeiss, Germany) at 10x objective magnification, an AxioCam MRc camera (Carl Zeiss, Germany) and AxioVision 4.6 software (Carl Zeiss, Germany). The wound closure areas were calculated at 12 h after incubation to show the closure of the wounds. Three independent experiments were photographed and quantified under a microscope. The data were normalized to the wound areas of the control (non-treated) cells.

### 4.12. Transwell Invasion Assay

The cells were pretreated with IM alone and in the presence of anti-FGF-2 or BGJ398 for 72 h, as shown for the wound-healing assay. The inserts containing 8-μm pores in 12-well Transwell plates (Greiner Bio-One, Austria) were coated with Matrigel (Corning Inc., Corning, NY, USA) and hardened for 24 h. Cells (5 × 10^5^) were placed in the upper inserts coated with Matrigel. Culture medium supplemented with 15% FBS was placed into the lower wells, whereas upper wells did not contain the growth factors (FBS—0%). After 24 h, the cells that migrated through the pores of inserts were fixed by 70% ethanol, stained with DAPI (Sigma-Aldrich, St. Louis, MO, USA), and counted by fluorescence microscopy Axiovert 200 (Carl Zeiss, Germany). Cells on the upper side of the membrane were carefully removed with cotton swabs. The numbers of invaded cells per field of view treated with IM alone or in presence of BGJ398 or anti-FGF-2 Abs were calculated. At least 9 fields in each case were counted. The experiments were performed in triplicate.

### 4.13. Colony Formation Assay

The cells were pretreated with IM alone and in the presence of anti-FGF-2 or BGJ398 for 72 h, as shown before. The cells were trypsinized, washed with PBS twice, and seeded for colony formation (approximately 500 cells for p100 culture dish) in complete DMEM-Hi medium with 10% FBS (Thermo Fisher Scientific, Waltham, MA, USA). The colonies were fixed by 70% ethanol, stained with Giemsa stain, and counted using Colony V1.1 software (Fujifilm, Japan) after 10 days of incubation. The experiments were performed in triplicate.

### 4.14. KIT/FGFR-2 Silencing Using siRNA

For FGFR2 and KIT knockdown experiments, On-Target Plus Smartpool siRNAs (Dharmacon, Lafayette, CO, USA) targeting *FGFR-2* (# L-003132-00-0005) or *KIT* (# L-003150-00-0005) were diluted in Opti-MEM (Thermo Fisher Scientific, Waltham, MA, USA) and complexed with RNAimax (Invitrogen, Carlsbad, CA, USA) to a final concentration of 40 nM oligonucleotides. The oligomer–RNAimax complex was added to the culture medium, and after 48–72 h, the knockdown was validated by immunoblotting.

### 4.15. RNA Extraction and Real-Time Quantitative PCR

Total RNA was extracted from GISTs and converted into cDNA, as previously described [15]. Briefly, 1 µL template cDNA was utilized for a real-time qPCR reaction with 5× qPCRmix-HS SYBR (PB025, Evrogen, Moscow, Russia) and forward and reverse primers (10 mM of each) for experimental or control genes. Real-time qPCR was performed by using the CFX96 Real-Time detection system (Bio-Rad, Hercules, CA, USA) according to the manufacturer’s protocol. The levels of each mRNA were normalized relative to GAPDH.

### 4.16. Enzyme-Linked Immunosorbent Assay

The levels of FGF2 in the serum of mice bearing IM-naive and -resistant GIST xenografts were measured using a human FGF2 ELISA Kit (R&D Systems, Tustin, CA, USA), according to the manufacturer’s protocol. The plates were read at 405 nm on a MultiScan FC plate reader (Thermo Fisher Scientific, Waltham, MA, USA).

### 4.17. Multiplex Analysis of Cytokines

A 41-multiplex analysis of an array of granules (MILLIPLEX MAP human cytokine/chemokine magnetic bead panel, Merck, Kenilworth, NJ, USA) was used to examine the levels of cytokines/chemokines in GIST supernatants. A MAGPIX^®^ reader (Bio-Rad, Hercules, CA, USA) was used with xPONENT software (Luminex Corp., Austin, Texas, USA). The Luminex MAGPIX^®^ instrument was previously calibrated with the MAGPIX^®^ Calibration Kit (EMD Millipore, Merck, Kenilworth, NJ, USA). The performance of the assay was verified with the MAGPIX^®^ Performance Verification Kit (EMD Millipore, Merck, Kenilworth, NJ, USA). MILLIPLEX^®^ analytes were used for counting the results and optimization of the performance of the MAGPIX reader.

### 4.18. Tumor Samples for KIT Mutational Analysis and Tissue Microarrays

A total of 30 patients diagnosed with GIST were enrolled in the present study, including 28 localized tumors from patients treated with primary complete surgery. The protocols for tumor sample collection and clinical record review were approved by the Ethics Committee of the Kazan State Medical University (IRB no. 98-0352B), and the patients provided informed written consent for the use of their tissues and clinical data in research. All personal information was removed. The slides were stained for hematoxylin/eosin and reviewed to confirm the diagnosis of GISTs; CD117 and DOG-1 expression were also evaluated by immunohistochemistry (IHC)-staining. In addition, the number of mitotic cells was calculated in 50 high-power fields (HPFs). Clinical risk score from primary GISTs was calculated according to the NIH GIST Consensus Criteria developed by Fletcher et al. [52] and classification of the risk categories proposed by the Armed Forces Institute of Pathology (AFIP) [53]. GIST specimens were examined for hot-spot mutation sites of *KIT* (exons 9, 11, 13, and 17). For this purpose, DNA was extracted from formalin-fixed, paraffin-embedded (FFPE) histological sections that were previously verified by pathologists (S.P. and E.M.). Tumor tissues were cut from the section and incubated in the extraction buffer (50 mmol/L KCl, 10 mmol/L Tris (pH8.3), 2.5 mmol/L MgCl_2_) supplemented with 60 μg/mL proteinase K for overnight at 55 °C. Proteinase K was inactivated by 10 min incubation at 95 °C. The lysates were centrifuged for 5 min and supernatants were used for PCR. Amplification of *c-KIT* was performed by PCR using specific oligonucleotide primers for exons 9, 11, 13, and 17 (Table S2) and a DNA Technology Termocycler, RF. PCR was carried out in 50 μL total reaction volume, including initial DNA denaturation at 94 °C for 15 min, 35 cycles each at 94 °C for 30 s, primer annealing at the temperature indicated (Table S3) for 30 s, elongation at 72 °C for 15 s, followed by one cycle at 72 °C for 2 min. PCR products were visualized by electrophoresis in 2% agarose, cut from the gel and purified by Wizard^®^ SV Gel and PCR Clean-Up System (Promega, Madison, WI, USA). Direct sequencing was performed by Applied Biosystems DNA analyzer with the same primers shown in the Table S2 and ABI PRISM^®^ BigDye™ Terminator v. 3.1 and an Applied Biosystems 3730 DNA Analyzer Sequencing ready reaction kit (Applied Biosystems, Foster City, CA, USA), according to the manufacturer’s protocol. DNA sequence alignment was performed with the NCBI Reference Sequence Database, employing online Alignment Search Tool BLAST [54] and Chromas SeqMan NGen version 12.0/Chromas (Technelysium Pty Ltd., South Brisbane, Australia) for multiple sequence alignment.

Formalin-fixed, paraffin-embedded tissue samples of representative tumor regions were used for the tissue microarray analysis (TMA). For this, the tissue cylinders (1.0 mm) were punched out from representative areas of each tissue block and transferred into a recipient paraffin block by using a manual tissue arrayer. Paraffin-embedded tissue sections were deparaffinized and rehydrated. Heat-induced epitope retrieval was applied before the staining with a primary antibody recognizing FGF-2, c-KIT, and DOG-1. Next, tissue microarrays were followed by a specific secondary antibody using the DAB Map detection kit (Ventana Medical Systems, Tucson, AR, USA). Tissue sections were also counterstained with hematoxylin and analyzed by light microscopy (Leica, Wetzlar, Germany). As mentioned before, all stained specimens were analyzed by 2 independent pathologists, S.P. and E.M.

### 4.19. GIST Xenograft Models

Subcutaneous tumor xenografts were established by s.c. inoculation of GIST cells (100 μL of 1 × 10^7^ cells/mL) into the flank areas of 5–8-week-old female nu/nu mice. The animal protocols were approved by the N.N. Blokhin National Medical Research Center of Oncology committee (des. 2019-1. from 15.01.2019) for Ethics of Animal Experimentation, and all the experiments were conducted in accordance with the Decision No. 81 of the Council of the Eurasian Economic Commission dated November 3, 2016 “On approval of Rules of good laboratory practice of the Eurasian Economic Union in the sphere of circulation of medicines” and Directive 2010/63/EU of the European Parliament and of the Council of 22 September 2010 on the protection of animals used for scientific purposes.

And all the experiments were conducted in accordance with the Guidelines for Animal Experiments in N.N. Blokhin National Medical Research Center of Oncology. GIST xenografts were allowed to reach an appropriate volume (~200 mm^3^) before the animals were randomized into the treatment groups (*n* = 4). Mice were orally administered either 50 μL of a vehicle (negative control), IM (50 mg/kg), BGJ398 (20 mg/kg), or a combination of the drugs indicated above. The changes in tumor volume, weight, and general health were recorded every second day. After the experiments reached to its end-point, the animals were sacrificed, tumors were excised and subjected to a histopathologic examination by 2 independent pathologists, S.P. and E.M. Formalin-fixed, paraffin-embedded (FFPE) tissues were sectioned at 4 μM for hematoxylin and eosin (H&E) stain and were also subjected to IHC-staining for FGF-2 expression. Histopathologic grading of the response to the treatment was also assessed by calculating the areas of necrosis, myxoid degeneration, or fibrosis, as previously described [41]. The images were captured using ScanScope XT (Aperio technologies inc., Vista, CA, USA).

### 4.20. Statistics

All the experiments were repeated a minimum of 3 times. The results are presented as the mean ± standard deviation (SD) for each group. Difference was considered significant at *p* < 0.05. The distribution of patients at risk of recurrence of GISTs was carried out according to the National Institute of Health (NIH) consensus criteria [52] and the Armed Forces Institute of Pathology (AFIP) criteria [53]. The relationship between FGF-2 expression and low/high risk of GIST patients was evaluated by drawing the receiver operating characteristic (AUROC) curve. The association between FGF-2 expression and patient’s groups was evaluated using the chi-square test or the Mann-Whitney U test.

## 5. Conclusions

We show here for the first time that IM induces profound changes in the secretome of IM-resistant GISTs, leading to the substantial production and release of chemokines, including FGF-2. This, in turn, activates the autocrine FGF2/FGFR-mediated loop in GISTs and promotes the malignant behavior of IM-resistant GISTs by facilitating their invasion, migration, and colony formation. The ability of IM to activate FGF2-mediated signaling in IM-resistant GISTs was revealed in vivo by using xenograft models and by assessing the expression of FGF-2 in tumor specimens of GIST patients who received IM-based therapy.

Taken together, the continuation of IM-based therapy for IM-resistant GISTs might have a negative impact and facilitate disease progression via the promotion of GIST malignant behavior in an FGF2-dependent manner. This provides a rationale to evaluate the effectiveness of the inhibitors of FGF-signaling for IM-resistant GISTs.

## Supplementary Materials

The following are available online at https://www.mdpi.com/2072-6694/12/6/1674/s1, Figure S1: The impact of inhibition of KIT signaling on growth kinetics and KIT expression of GIST cells. Figure S2: FGF-2 stimulates invasion, migration, and colony formation of IM-resistant GIST cells. Figure S3: The effects of BGJ398 and anti-FGF-2 Abs on invasion, migration and colony formation of IM-treated GIST 430 cells. Figure S4: Co-immunoprecipitation of KIT and FGFR1 or 2 in IM-resistant GIST. Figure S5: A cross-talk between FGFR1 and KIT in IM-resistant GIST. Figure S6: Changes in growth kinetics of GIST T-1 cells treated with DMSO (negative control), FGF-2 (100 ng/mL) (positive control), IM (0.25 µmol/L) alone or in presence of FGF-2. Figure S7: Whole western blot for Figure 3A. Figure S8: Whole western blot for Figure 3C. Figure S9: Whole western blot for Figure S4. Table S1: The characteristics of GIST patients enrolled in present study. Table S2: IHC-staining scale for KIT/FGF-2 in GIST specimens. Table S3: PCR oligonucleotide primers and reaction conditions for amplification and direct sequencing of c-KIT (exons 9, 11, 13 and 17) and PDGFRA (exons 12 and 18).

## Statement of Significance

As secondary KIT mutations are known as a common mechanism of acquired resistance to imatinib mesylate (IM) in GISTs, the alternative molecular mechanisms were also described. For example, we have shown previously that the activation of FGF-signaling is responsible for GIST resistance to IM, whereas the targeting of FGF-signaling effectively re-sensitizes tumors to IM. We show here for the first time that IM induces the activation of the FGF2/FGFR autocrine loop in IM-resistant GISTs both in vitro and in vivo, whereas the inhibition of FGF-signaling has a strong inhibitory impact on the malignant behavior and growth of IM-resistant GISTs both in vitro and in vivo. Continuation of IM-based therapy for GIST patients with secondary resistance to IM might have a negative impact on disease progression due to the activation of FGF-signaling, thereby providing a rationale to evaluate the effectiveness of FGFR-inhibitors to improve the therapeutic strategies for IM-resistant GISTs.

## References

[1] Hirota S. (1998). Gain-of-Function Mutations of c-kit in Human Gastrointestinal Stromal Tumors. Science.

[2] Rubin B.P., Singer S., Tsao C., Duensing A., Lux M.L., Ruiz R., Hibbard M.K., Chen C.J., Xiao S., Tuveson D.A. (2001). KIT activation is a ubiquitous feature of gastrointestinal stromal tumors. Cancer Res..

[3] Heinrich M.C., Corless C.L., Duensing A., McGreevey L., Chen C.J., Joseph N., Singer S., Griffith D.J., Haley A., Town A. (2003). PDGFRA activating mutations in gastrointestinal stromal tumors. Science.

[4] Tuveson D., Willis N.A., Jacks T., Griffin J.D., Singer S., Fletcher C.D., A Fletcher J., Demetri G.D. (2001). STI571 inactivation of the gastrointestinal stromal tumor c-KIT oncoprotein: Biological and clinical implications. Oncogene.

[5] Demetri G.D., Von Mehren M., Blanke C.D., Abbeele A.D.V.D., Eisenberg B., Roberts P.J., Heinrich M.C., Tuveson D., Singer S., Janicek M.J. (2002). Efficacy and Safety of Imatinib Mesylate in Advanced Gastrointestinal Stromal Tumors. N. Engl. J. Med..

[6] Verweij J., Casali P.G., Zalcberg J., LeCesne A., Reichardt P., Blay J.Y., Issels R., Van Oosterom A., Hogendoorn P.C., Van Glabbeke M. (2004). Progression-free survival in gastrointestinal stromal tumors with high-dose imatinib: Randomized trial. Lancet.

[7] Gramza A.W., Corless C.L., Heinrich M.C. (2009). Resistance to Tyrosine Kinase Inhibitors in Gastrointestinal Stromal Tumors. Clin. Cancer Res..

[8] Rock E.P., Goodman V., Jiang J.X., Mahjoob K., Verbois S.L., Morse D., Dagher R., Justice R., Pazdur R. (2007). Food and Drug Administration Drug Approval Summary: Sunitinib Malate for the Treatment of Gastrointestinal Stromal Tumor and Advanced Renal Cell Carcinoma. Oncologist.

[9] Demetri G.D., Reichardt P., Kang Y.-K., Blay J.-Y., Rutkowski P., Gelderblom H., Hohenberger P., Leahy M., Von Mehren M., Joensuu H. (2012). Efficacy and safety of regorafenib for advanced gastrointestinal stromal tumours after failure of imatinib and sunitinib (GRID): An international, multicentre, randomised, placebo-controlled, phase 3 trial. Lancet.

[10] Miselli F., Casieri P., Negri T., Orsenigo M., Lagonigro M.S., Gronchi A., Fiore M., Casali P.G., Bertulli R.M., Carbone A. (2007). c-Kit/PDGFRA Gene Status Alterations Possibly Related to Primary Imatinib Resistance in Gastrointestinal Stromal Tumors. Clin. Cancer Res..

[11] Sakurama K., Noma K., Takaoka M., Tomono Y., Watanabe N., Hatakeyama S., Ohmori O., Hirota S., Motoki T., Shirakawa Y. (2009). Inhibition of focal adhesion kinase as a potential therapeutic strategy for imatinib-resistant gastrointestinal stromal tumor. Mol. Cancer Ther..

[12] Tarn C., Rink L., Merkel E., Flieder U., Pathak H., Koumbi D., Testa J.R., Eisenberg B., Von Mehren M., Godwin A.K. (2008). Insulin-like growth factor 1 receptor is a potential therapeutic target for gastrointestinal stromal tumors. Proc. Natl. Acad. Sci. USA.

[13] Agaram N.P., Wong G.C., Guo T., Maki R.G., Singer S., DeMatteo R.P., Besmer P., Antonescu C.R. (2008). Novel V600E BRAF mutations in imatinib-naive and imatinib-resistant gastrointestinal stromal tumors. Genes Chromosom. Cancer.

[14] Mahadevan D., Cooke L., Riley C., Swart R., Simons B., Della Croce K., Wisner L., Iorio M., Shakalya K., Garewal H. (2007). A novel tyrosine kinase switch is a mechanism of imatinib resistance in gastrointestinal stromal tumors. Oncogene.

[15] Boichuk S.V., Galembikova A., Dunaev P., Valeeva E., Elena S., Gusev O., Khaiboullina S.F. (2017). A Novel Receptor Tyrosine Kinase Switch Promotes Gastrointestinal Stromal Tumor Drug Resistance. Molecules.

[16] Boichuk S.V., Galembikova A., Dunaev P., Micheeva E., Valeeva E., Novikova M., Khromova N., Kopnin P. (2018). Targeting of FGF-Signaling Re-Sensitizes Gastrointestinal Stromal Tumors (GIST) to Imatinib In Vitro and In Vivo. Molecules.

[17] Li F., Huynh H., Li X., Ruddy D., Wang Y., Ong R., Chow P., Qiu S., Tam A., Rakiec D.P. (2015). FGFR-Mediated Reactivation of MAPK Signaling Attenuates Antitumor Effects of Imatinib in Gastrointestinal Stromal Tumors. Cancer Discov..

[18] Javidi-Sharifi N., Traer E., Martinez J., Gupta A., Taguchi T., Dunlap J., Heinrich M.C., Corless C.L., Rubin B.P., Druker B.J. (2014). Crosstalk between KIT and FGFR3 Promotes Gastrointestinal Stromal Tumor Cell Growth and Drug Resistance. Cancer Res..

[19] Kelly C., Shoushtari A.N., Qin L.-X., D’Angelo S.P., Dickson M.A., Gounder I.M., Keohan M.L., McFadyen C., Sjoberg A., Singer S. (2018). A phase Ib study of BGJ398, a pan-FGFR kinase inhibitor in combination with imatinib in patients with advanced gastrointestinal stromal tumor. Investig. New Drugs.

[20] Taguchi T., Sonobe H., Toyonaga S.-I., Yamasaki I., Shuin T., Takano A., Araki K., Akimaru K., Yuri K. (2002). Conventional and molecular cytogenetic characterization of a new human cell line, GIST-T1, established from gastrointestinal stromal tumor. Lab. Investig..

[21] Bauer S., Duensing A., Demetri G.D., Fletcher J.A. (2007). KIT oncogenic signaling mechanisms in imatinib-resistant gastrointestinal stromal tumor: PI3-kinase/AKT is a crucial survival pathway. Oncogene.

[22] Gaubert F., Escaffit F., Bertrand C., Korc M., Pradayrol L., Clemente F., Estival A. (2001). Expression of the high molecular weight fibroblast growth factor-2 isoform of 210 amino acids is associated with modulation of protein kinases C delta and epsilon and ERK activation. J. Biol. Chem..

[23] Arnaud E., Touriol C., Boutonnet C., Gensac M.C., Vagner S., Prats H., Prats A.C. (1999). A new 34-kilodalton isoform of human fibroblast growth factor 2 is cap dependently synthesized by using a non-AUG start codon and behaves as a survival factor. Mol. Cell Biol..

[24] Timmer M., Müller-Ostermeyer F., Kloth V., Winkler C., Grothe C., Nikkhah G. (2004). Enhanced survival, reinnervation, and functional recovery of intrastriatal dopamine grafts co-transplanted with Schwann cells overexpressing high molecular weight FGF-2 isoforms. Exp. Neurol..

[25] Korc M., Friesel R.E. (2009). The role of fibroblast growth factors in tumor growth. Curr. Cancer Drug Targets.

[26] Giavazzi R., Sennino B., Coltrini D., Garofalo A., Dossi R., Ronca R., Tosatti M.P.M., Presta M. (2003). Distinct Role of Fibroblast Growth Factor-2 and Vascular Endothelial Growth Factor on Tumor Growth and Angiogenesis. Am. J. Pathol..

[27] Lefevre G., Babchia N., Calipel A., Mouriaux F., Faussat A.M., Mrzyk S., Mascarelli F. (2009). Activation of the FGF2/FGFR1 autocrine loop for cell proliferation and survival in uveal melanoma cells. Invest. Ophthalmol. Vis. Sci..

[28] Marek L., Ware K.E., Fritzsche A., Hercule P., Helton W.R., Smith J.E., McDermott L.A., Coldren C.D., Nemenoff R.A., Merrick D.T. (2008). Fibroblast Growth Factor (FGF) and FGF Receptor-Mediated Autocrine Signaling in Non-Small-Cell Lung Cancer Cells. Mol. Pharmacol..

[29] Feng S., Wang J., Zhang Y., Creighton C., Ittmann M. (2015). FGF23 promotes prostate cancer progression. Oncotarget.

[30] Terai H., Soejima K., Yasuda H., Nakayama S., Hamamoto J., Arai D., Ishioka K., Ohgino K., Ikemura S., Sato T. (2013). Activation of the FGF2-FGFR1 Autocrine Pathway: A Novel Mechanism of Acquired Resistance to Gefitinib in NSCLC. Mol. Cancer Res..

[31] Ware K.E., Hinz T.K., Kleczko E., Singleton K.R., Marek L.A., A Helfrich B., Cummings C.T., Graham D.K., Astling D., Tan A.-C. (2013). A mechanism of resistance to gefitinib mediated by cellular reprogramming and the acquisition of an FGF2-FGFR1 autocrine growth loop. Oncogenesis.

[32] Bossard C., Laurell H., Van den Berghe L., Meunier S., Zanibellato C., Prats H. (2003). Translokin is an intracellular mediator of FGF-2 trafficking. Nat. Cell Biol..

[33] Van den Berghe L., Laurell H., Huez I., Zanibellato C., Prats H., Bugler B. (2000). FIF [Fibroblast growth factor-2(FGF-2)-interacting-factor], a nuclear putatively antiapoptotic factor, interacts specifically with FGF-2. Mol. Endocrinol..

[34] Coleman S.J., Chioni A.-M., Ghallab M., Anderson R.K., Lemoine N., Kocher H.M., Grose R.P. (2014). Nuclear translocation of FGFR 1 and FGF 2 in pancreatic stellate cells facilitates pancreatic cancer cell invasion. EMBO Mol. Med..

[35] Tanner Y., Grose R. (2016). Dysregulated FGF signaling in neoplastic disorders. Semin. Cell Dev. Biol..

[36] Wang K., Ji W., Yu Y., Li Z., Niu X., Xia W., Lu S. (2018). FGFR1-ERK1/2-SOX2 axis promotes cell proliferation, epithelial–mesenchymal transition, and metastasis in FGFR1-amplified lung cancer. Oncogene.

[37] Knuchel S., Anderle P., Werfelli P., Diamantis E., Rüegg C. (2015). Fibroblast surface-associated FGF-2 promotes contact-dependent colorectal cancer cell migration and invasion through FGFR-SRC signaling and integrin αvβ5-mediated adhesion. Oncotarget.

[38] Santolla M.F., Vivacqua A., Lappano R., Rigiracciolo D.C., Cirillo F., Galli G.R., Talia M., Brunetti G., Miglietta A.M., Belfiore A. (2019). GPER mediates a feedforward FGF2/FGFR1 paracrine activation coupling CAFs to cancer cells toward breast tumor progression. Cells.

[39] Huang E., Hynes M.J., Zhang T., Ginestier C., Dontu G., Appelman H., Fields J.Z., Wicha M.S., Boman B.M. (2009). Aldehyde dehydrogenase 1 is a marker for normal and malignant human colonic stem cells (SC) and tracks SC overpopulation during colon tumorigenesis. Cancer Res..

[40] Kostas M., Lampart A., Bober J., Wiedlocha A., Tomala J., Krowarsch D., Otlewski J., Zakrzewska M. (2018). Translocation of Exogenous FGF1 and FGF2 Protects the Cell against Apoptosis Independently of Receptor Activation. J. Mol. Boil..

[41] Zhen Y., Sorensen V., Skjerpen C.S., Haugsten E.M., Jin Y., Wälchli S. (2012). Nuclear import of exogenous FGF1 requires the ER-protein LRRC59 and the importins Kpnalpha1 and Kpnbeta1. Traffic.

[42] Takahashi M., Fukuoka M., Yoshioka K., Hohjoh H. (2016). Neighbors’ death is required for surviving human adenocarcinoma PC-9 cells in an early stage of gefitinib treatment. Biochem. Biophys. Res. Commun..

[43] Fukuoka M., Yoshioka K., Hohjoh H. (2018). NF-κB activation is an early event of changes in gene regulation for acquiring drug resistance in human adenocarcinoma PC-9 cells. PLoS ONE.

[44] Song S., Wientjes M.G., Gan Y., Au J.L. (2000). Fibroblast growth factors: An epigenetic mechanism of broad spectrum resistance to anticancer drugs. Proc. Natl. Acad. Sci. USA.

[45] Menzel T., Rahman Z., Calleja E., White K., Wilson E.L., Wieder R., Gabrilove J. (1996). Elevated intracellular level of basic fibroblast growth factor correlates with stage of chronic lymphocytic leukemia and is associated with resistance to fludarabine. Blood.

[46] Ruotsalainen T., Joensuu H., Mattson K., Salven P. (2002). High pretreatment serum concentration of basic fibroblast growth factor is a predictor of poor prognosis in small cell lung cancer. Cancer Epidemiol. Biomark. Prev..

[47] Salven P., Orpana A., Teerenhovi L., Joensuu H. (2000). Simultaneous elevation in the serum concentrations of the angiogenic growth factors VEGF and bFGF is an independent predictor of poor prognosis in non-Hodgkin lymphoma: A single-institution study of 200 patients. Blood.

[48] Leunig A., Tauber S., Spaett R., Grevers G., Leunig M. (1998). Basic fibroblast growth factor in serum and urine of patients with head and neck cancer. Oncol. Rep..

[49] Dirix L.Y., Vermeulen P.B., Hubens G., Benoy I., Martin M., De Pooter C., Van Oosterom A.T. (1996). Serum basic fibroblast growth factor and vascular endothelial growth factor and tumour growth kinetics in advanced colorectal cancer. Ann. Oncol..

[50] Massari F., Ciccarese C., Santoni M., Lopez-Beltran A., Scarpelli M., Montironi R., Cheng L. (2015). Targeting fibroblast growth factor receptor (FGFR) pathway in renal cell carcinoma. Expert Rev. Anticancer Ther..

[51] Costes S.V., Daelemans D., Cho E.H., Dobbin Z., Pavlakis G., Lockett S. (2004). Automatic and quantitative measurement of protein-protein colocalization in live cells. Biophys. J..

[52] Fletcher C.D., Berman J.J., Corless C., Gorstein F., Lasota J., Longley B.J., Miettinen M., O’Leary T.J., Remotti H., Rubin B.P. (2002). Diagnosis of gastrointestinal stromal tumors: A consensus approach. Int. J. Surg. Pathol..

[53] Miettinen M., Lasota J. (2006). Gastrointestinal stromal tumors: Pathology and prognosis at different sites. Semin. Diagn. Pathol..

[54] The NCBI Reference Sequence Database, employing online Alignment Search Tool BLAST. https://blast.ncbi.nlm.nih.gov/.

