# Supplementary material for: Inhibition of FGF2-Mediated Signaling in GIST—Promising Approach for Overcoming Resistance to Imatinib
Source: Cancers (Basel). 2020 Jun 24;12(6):1674. doi: 10.3390/cancers12061674 (PMC7352302; doi:10.3390/cancers12061674)
Supplement: Supplementary file 1 [file cancers-12-01674-s001.pdf]

cancers
MDPI

Supplementary Materials: Inhibition of FGF2-Mediated Signaling in GIST—Promising Approach for Overcoming Resistance to Imatinib

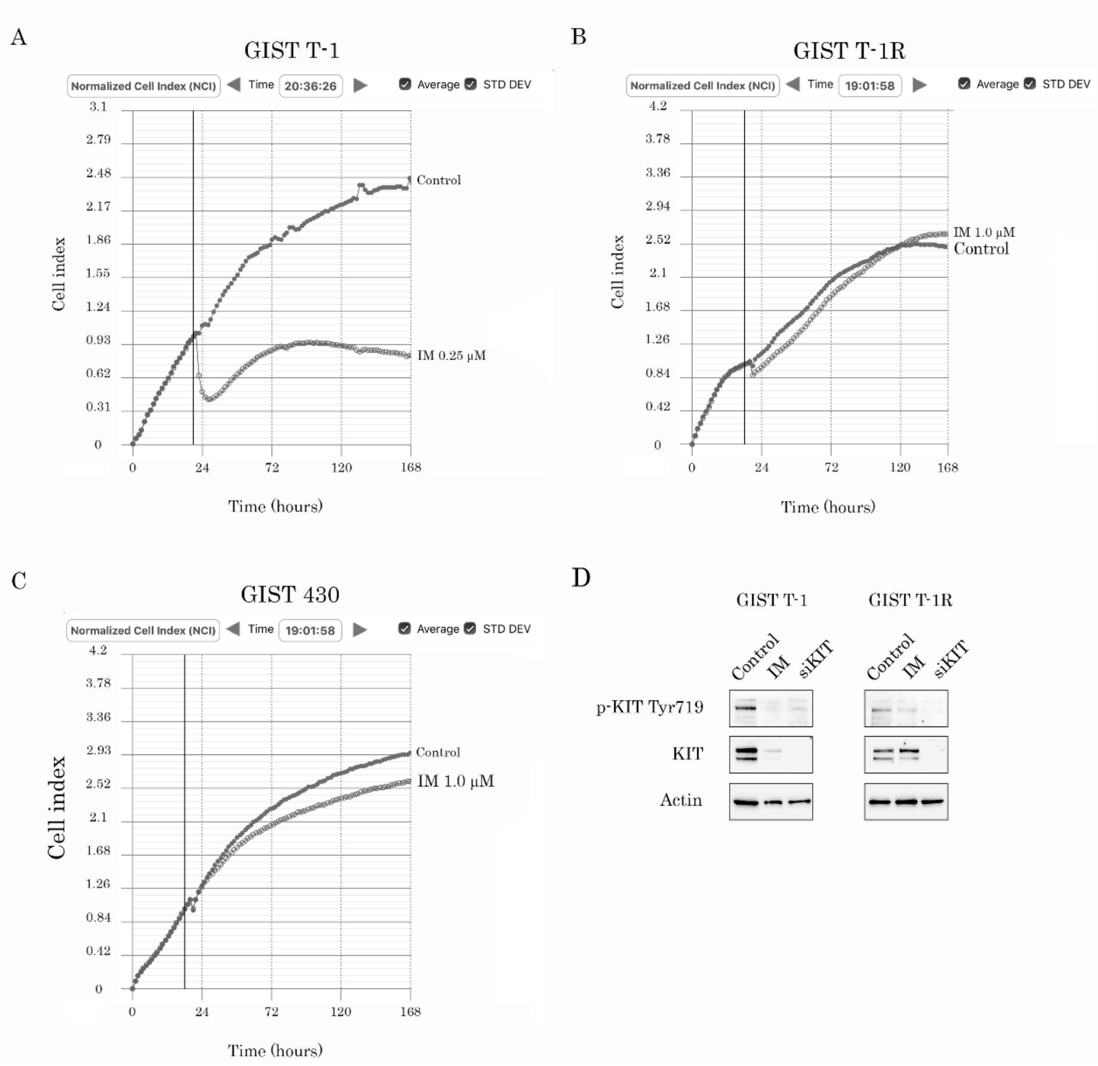

Figure S1. The impact of inhibition of KIT signaling on growth kinetics and KIT expression of GIST cells. (**A**–**C**) Changes in growth kinetics of GIST T-1 (**A**), GIST T-1R (**B**), and GIST 430 (**C**) cells treated with DMSO (negative control) or IM. (**D**) Inhibition of KIT phosphorylation and total KIT expression in GIST T-1 vs. T-1R cells treated with IM or siRNA *KIT*. Actin was used for the loading control.

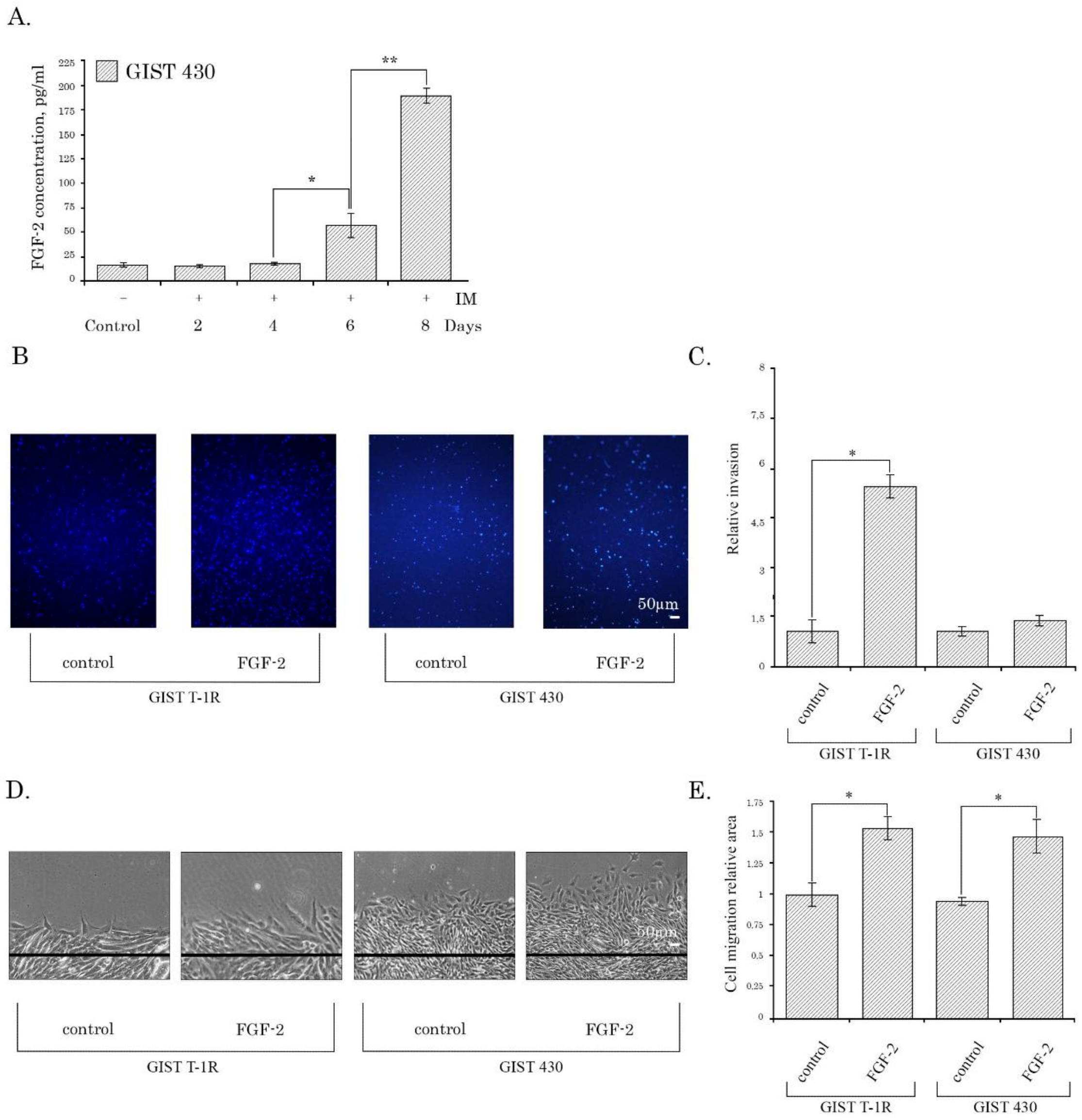

Figure S2. FGF-2 stimulates invasion, migration, and colony formation of IM-resistant GIST cells. (**A**) FGF-2 production by GIST 430 cells treated with IM (1 μmol/L) for 2-8 days. (**B**) Representative images of transwell invasion assay illustrating the effect of FGF-2 on the invasion of GIST T-1R (left) and GIST 430 (right) cells. The cells were treated with FGF-2 (20 ng/mL) for 5 days. (**C**) Quantitative analysis of FGF-2-induced invasion of IM-resistant GIST cells. Columns, mean of at least 3 independent experiments; bars, SE. (**D**) Representative images of wound-healing assay illustrating the effect of FGF-2 on the migration of GIST T-1R (left) and GIST 430 (right) cells. The cells were treated with FGF-2 (20 ng/mL) for 5 days. (**E**) Quantitative analysis of FGF-2-induced migration of IM-resistant GIST cells. Columns, mean of at least 3 independent experiments; bars, SD. Significant differences with $p < 0.05$ (*), $p < 0.01$ (**) from $n \geq 3$ using unpaired Student's t-test.

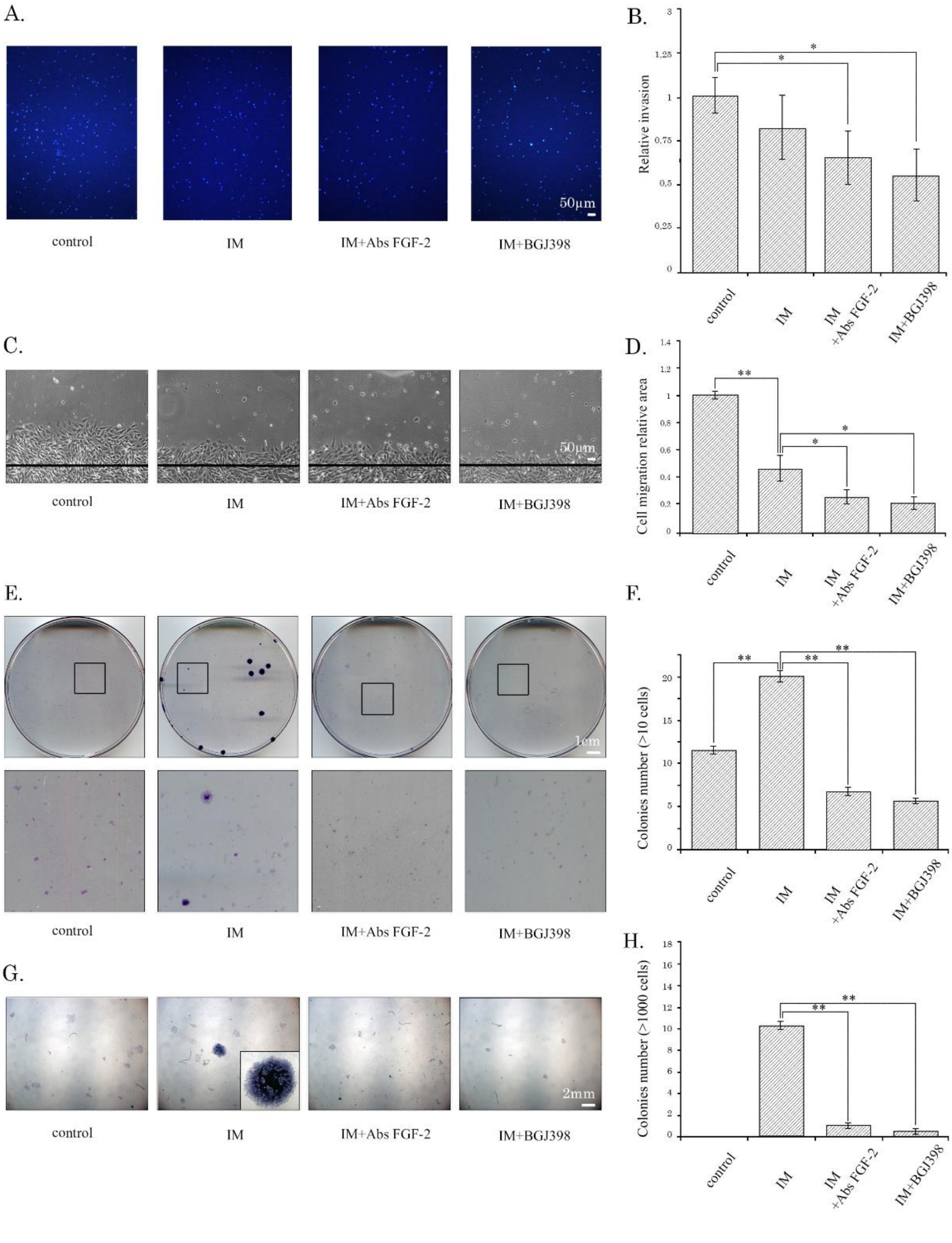

Figure S3. The effects of BGJ398 and anti-FGF-2 Abs on invasion, migration and colony formation of IM-treated GIST 430 cells. (**A**) Matrigel transwell invasion assay representative images (20×) of GIST 430 cells treated with vehicle, IM alone or in presence of BGJ398 (1 μmol/L), a selective FGFR inhibitor, or anti-FGF-2 neutralizing Abs (20 μg/mL). (**B**) Transwell invasion assay quantification as the number of invading GIST 430 cells per microscopic field after treatment with vehicle, IM or IM in presence of BGJ398 or anti-FGF-2 Abs; (**C**) Representative images (20x) of the wound healing assay of GIST 430 cells upon IM treatment (1 μmol/L) for 48 h alone or in presence of BGJ398, a selective FGFR-inhibitor (1 μmol/L) or anti-FGF2 neutralizing Abs (20 μg/mL). GIST cells treated with vehicle were used as control. (**D**) Quantification of wound area in control, FGF-2 or IM-treated GIST 430 cells; (**E**) Representative images of the colony formation assay of GIST 430 cells (20x) upon IM treatment (1 μmol/L) for 48 h alone or in presence of BGJ398(1 μmol/L) or anti-FGF-2 neutralizing Abs (20 μg/mL).

GIST cells treated with vehicle were used as control. (**F**) Quantification of colonies numbers in control or IM-treated GIST 430 cells alone or in presence of BGJ398 or anti-FGF-2 Abs. Data are presented as median ± SD. (**G**) The representative images of large-size spherical colonies formed by GIST 430 cells treated with IM alone or in presence of BGJ398 or anti-FGF-2 Abs. (H) Quantification of large-size spheroids in control, IM-treated GIST 430 cells in absence or presence of BGJ398 or anti-FGF-2 Abs. Data are presented as median ± SD. Significant differences with $p < 0.05$ (*), $p < 0.01$ (**) from $n \geq 3$ using unpaired Student's t-test.

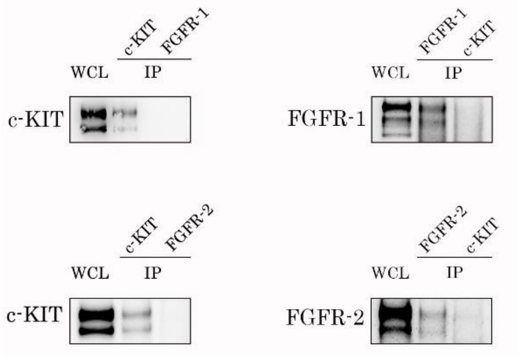

Figure S4. Co-immunoprecipitation of KIT and FGFR1 or 2 in IM-resistant GIST. Left panel -KIT expression in GIST cell lysates immunoprecipitated by anti-KIT or anti-FGFR-1 (up) – FGFR-2 (bottom) Abs to demonstrate endogenous complex formation. A whole-cell lysate (WCL) illustrating KIT expression is included; Right panel - Expression of FGFR1 (up), or FGFR-2 (bottom) in IM-resistant GIST cell lysates immunoprecipitated by anti-KIT or anti-FGFR1 (up), anti-FGFR-2 (bottom) Abs. A whole-cell lysates (WCL) illustrating expression of FGFR-1 or 2 were included.

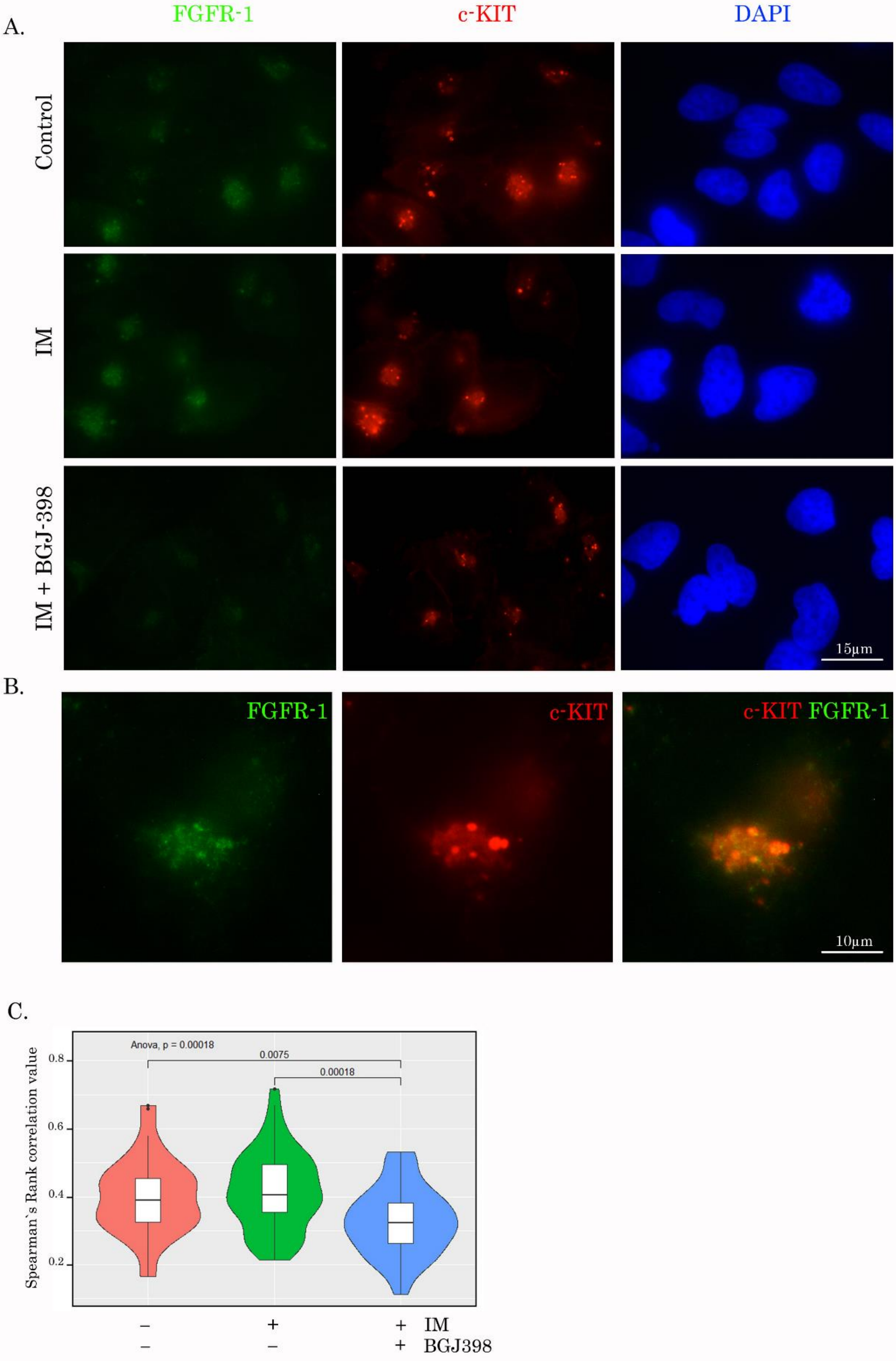

Figure S5. A cross-talk between FGFR1 and KIT in IM-resistant GIST. (**A**) GIST T-1R cells were treated with IM (1 μmol/L) for 48 h alone (middle panel) or in presence of BGJ398, a selective FGFR inhibitor (bottom panel), and followed by double immunofluorescence staining for FGFR1 and KIT. Images were also merged with DAPI staining to outline the nucleus. (**B**) 100x immunofluorescence image

illustrating the co-localization pattern between FGFR1 (green) and KIT (red) in GIST T-1R cells. (**C**) Graph illustrating the distributions of Spearman's rank correlation values between GIST T1-R cells treated with DMSO (control) (mean = 0.4), IM alone (mean = 0.42) or in presence of BGJ398 (mean = 0.33). p values are shown on the figure.

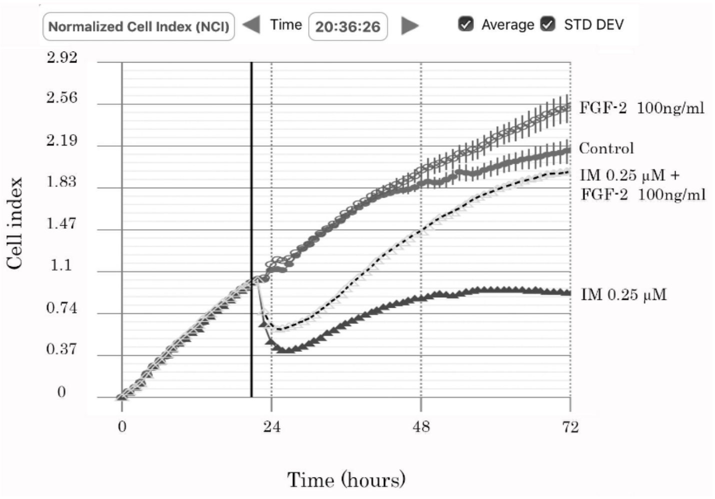

Figure S6. Changes in growth kinetics of GIST T-1 cells treated with DMSO (negative control), FGF-2 (100 ng/mL) (positive control), IM (0.25 μmol/L) alone or in presence of FGF-2.

Whole Blot (Uncropped Blots)

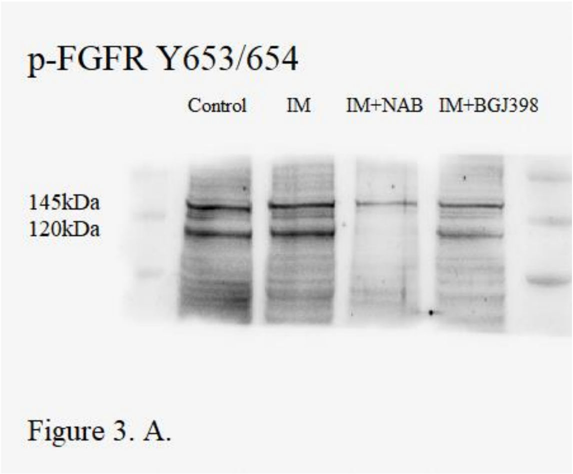

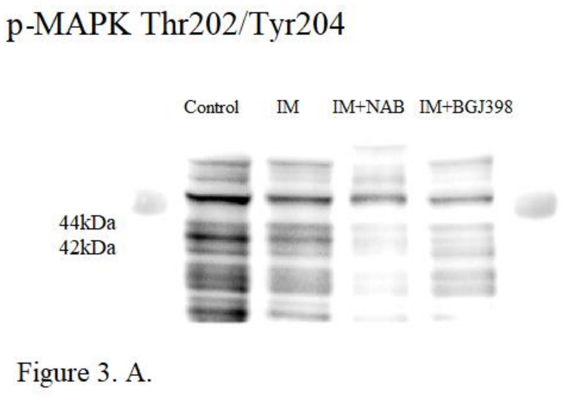

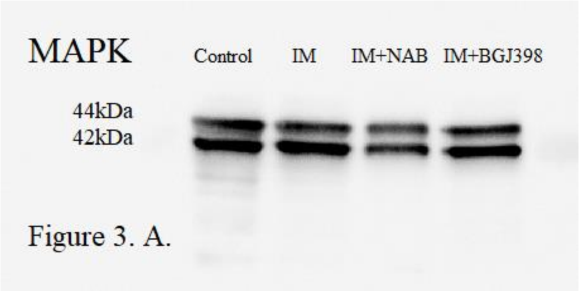

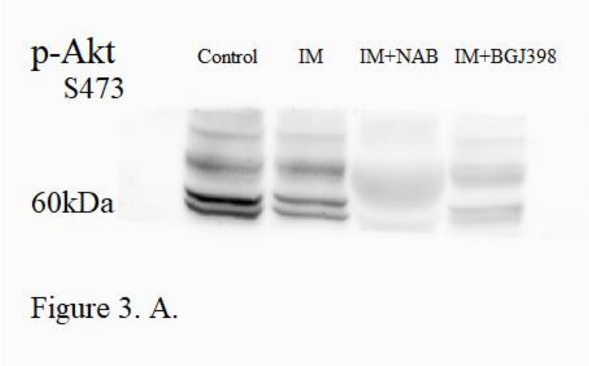

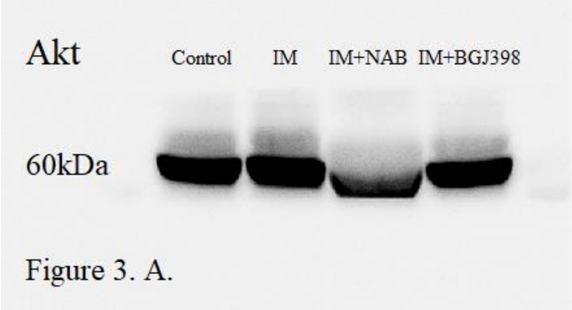

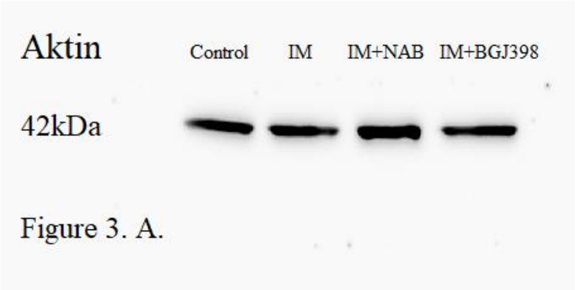

Figure S7. Whole western blot for figure 3A.

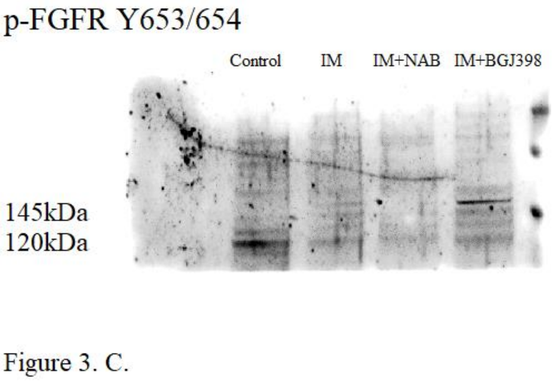

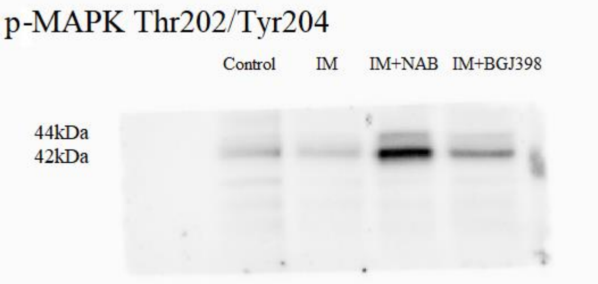

Figure 3. C.

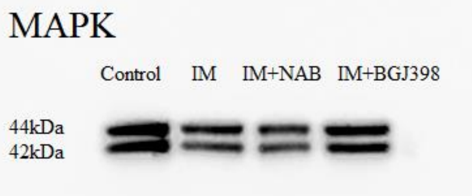

Figure 3. C.

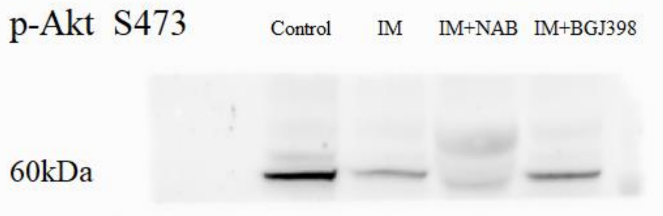

Figure 3. C.

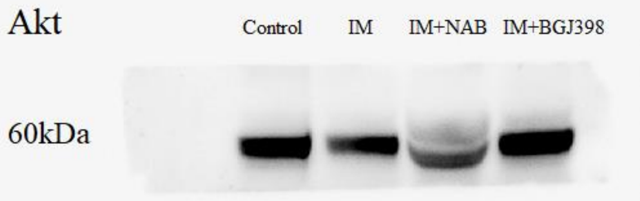

Figure 3. C.

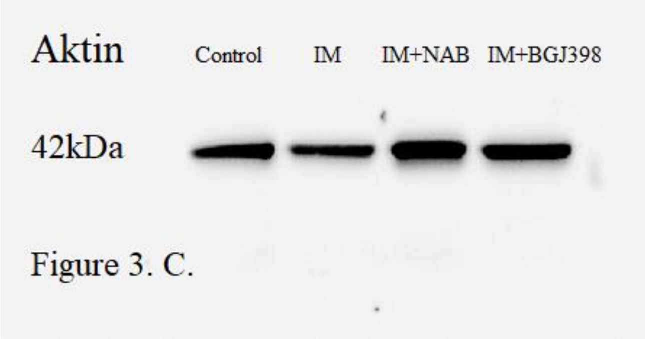

Figure S8. Whole western blot for figure 3C.

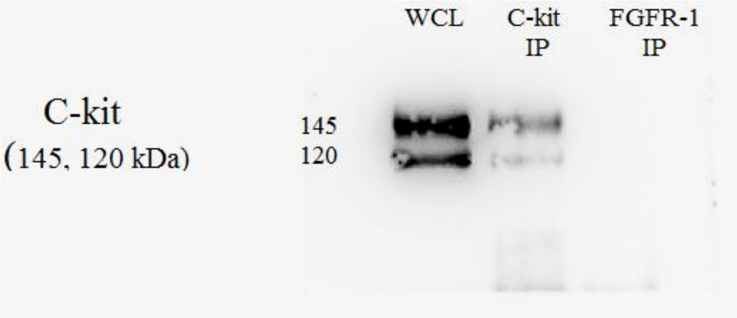

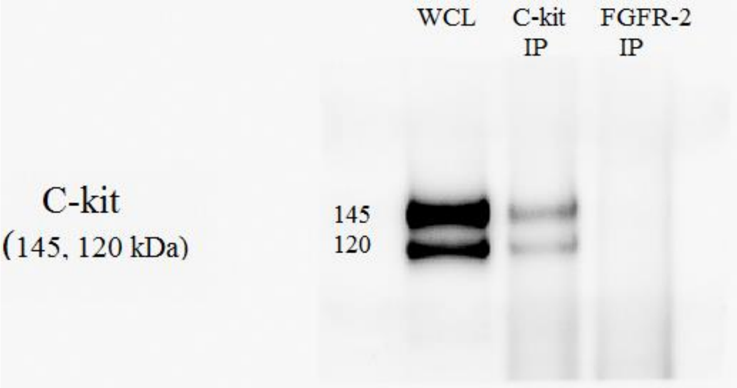

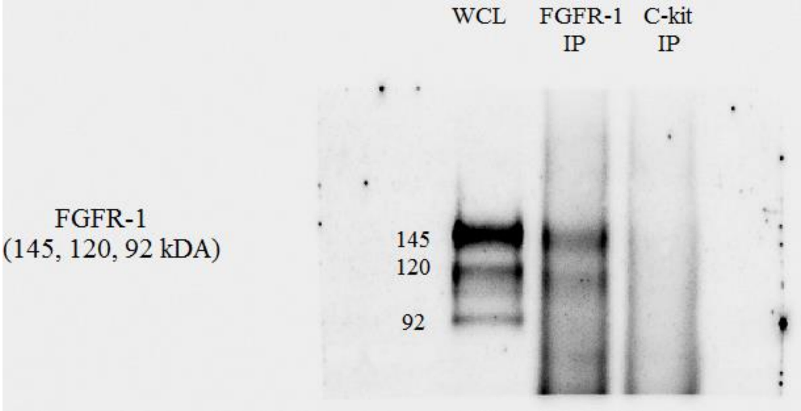

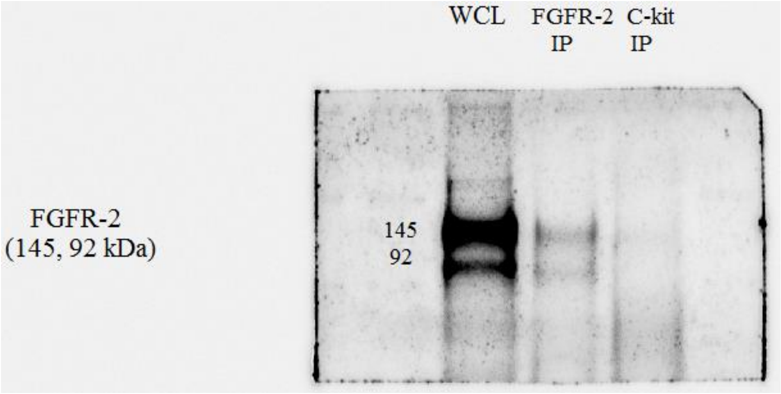

Figure S9. Whole western blot for figure S4.

Table S1. The characteristics of GIST patients enrolled in present study.

Case #	Age	Gender	Location	Size [cm]	Risk	*KIT*/*PDGFRA* Mutation Status	Type of Mutation	Histological Type	CD117	FGF-2	IM Treatment
1	55	M	stomach	8	NA	*KIT* Exon 11	V559D	spindle-cell	NA	4	+
2	63	F	ovary (m)	NA	NA	*KIT* Exon 11	V560D	spindle-cell	NA	2	+
3	78	F	small bowel (r)	6.2	high	WT		epithelioid	3	2	+
4	64	M	greater omentum (m)	3.4	high	*KIT* Exon 11	Del. V559 -E561 (homo)	epithelioid	2	2	+
5	57	M	small bowel (r)/pelvic cavity (m)	10/1.1	high	*KIT* Exon 11	D572Y	epithelioid	2	3	+
6	63	M	rectum	5	high	*KIT* Exon 17	C809R	NA	2	0	+
7	65	F	stomach	7.3	low	WT		epithelioid	2	2	+
8	59	M	small bowel	14.2	high	*KIT* Exon 11	V560A	epithelioid	2	NA	+
9	51	M	small bowel	12	high	*KIT* Exon 9	Dupl. A502 Y503	epithelioid	2	3	+
10	60	M	small bowel	7	high	*KIT* Exon 11	V559D	mixed	3	2	+
11	59	F	small bowel	10	high	*KIT* Exon 11	V560D	spindle-cell	2	0	-
12	56	F	small bowel	3	low	*KIT* Exon 13	P627A	spindle-cell	1	0	-
13	55	F	small bowel	10	high	WT		spindle-cell	0	0	-
14	61	F	stomach	7.3	high	*KIT* Exon 13	G648S	spindle-cell	2	0	-
15	59	M	stomach	22.5	high	*KIT* Exon 11	Del. K558-P585 (28 a/o)	epithelioid	2	0	-
16	60	F	stomach	2	low	*KIT* Exon 11	W557R	spindle-cell	3	0	-
17	59	F	stomach	17	high	WT		mixed	1	0	-
18	74	M	stomach	5	high	WT		epithelioid	2	0	-
19	67	F	stomach	5	high	*KIT* Exon 9	N511D (homo)	epithelioid	0	1	-
20	53	F	stomach	3	low	WT		mixed	0	3	-
21	67	F	stomach	12.3	high	*KIT* Exon 9	A482T	mixed	2	1	-
22	69	F	stomach	1.5	low	*KIT* Exon 11	R586I	epithelioid	2	3	-

23	69	F	stomach	1.5	low	*KIT* Exon 11	Dupl. L567 P568 Y569 D570	spindle-cell	2	3	-
24	65	F	stomach	0.8	low	WT		mixed	2	2	-
25	56	F	stomach	3	low	WT		spindle-cell	2	3	-
26	57	F	stomach	4.4	low	*KIT* Exon 11	V559D	spindle-cell	2	4	-
27	61	F	stomach	17/9	high	*KIT* Exon 11	V559D	spindle-cell	1	1	-
28	65	F	stomach	1,5	low	*PDGFRA* Exon 18	D842E	mixed	3	NA	-
29	54	M	stomach	6	high	*KIT* Exon 11	R586I	epithelioid	1	NA	-
30	60	M	stomach	4	low	*KIT* Exon 11	V559D	epithelioid	2	0	-

Table S2. IHC-staining scale for KIT/FGF-2 in GIST specimens.

KIT	FGF-2
0—negative	0—negative
1—moderate	1—faint (membranous)
2—intermediate	2—faint (nuclear)
3—bright	3—intermediate (nuclear)
	4—bright (nuclear)

Table S3. PCR oligonucleotide primers and reaction conditions for amplification and direct sequencing of c-KIT (exons 9, 11, 13 and 17) and PDGFRA (exons 12 and 18).

Primers	Primer Sequence, 5′–3′	Annealing Temperature, °C
C-KIT ex9 For	AAAAGTATGCCACATCCCAGGTG	60
C-KIT ex9 Rev	AGAAATGATATGGTCAATGTTGG	
C-KIT ex11 For	TAGCTGGCATGATGTGCATT	58
C-KIT ex11 Rev	TGGAAAGCCCCTGTTTCATA	
C-KIT ex13 For	CTTAACTTGTTGTCTTCCTTC	58
C-KIT ex13 Rev	CCAAGCAGTTTATAATCTAGCA	
C-KIT ex17 For	GTTTTCTTTTCTCCTCCAACCTA	60
C-KIT ex17 Rev	CCTAGACAGGATTTACATTATGA	
PDGFRA ex12 For	TCC AGT CAC TGT GCT GCT TC	62
PDGFRA ex12 Rev	GCA AGG GAA AAG GGA GTC TT	60
PDGFRA ex18 For	TTC CTT TTC CAT GCA GTG TGT CC	68
PDGFRA ex18 Rev	GAA GCA ACA CCT GAC TTT AGA GA	66

© 2019 by the authors. Licensee MDPI, Basel, Switzerland. This article is an open access article distributed under the terms and conditions of the Creative Commons Attribution (CC BY) license (http://creativecommons.org/licenses/by/4.0/).
